# Brief Review of PVDF Properties and Applications Potential

**DOI:** 10.3390/polym14224793

**Published:** 2022-11-08

**Authors:** Rashid Dallaev, Tatiana Pisarenko, Dinara Sobola, Farid Orudzhev, Shikhgasan Ramazanov, Tomáš Trčka

**Affiliations:** 1 Department of Physics, Faculty of Electrical Engineering and Communication, Brno University of Technology, Technická 2848/8, 61600 Brno, Czech Republic; 2 Central European Institute of Technology, Purkyňova 656/123, 61200 Brno, Czech Republic; 3 Immanuel Kant Baltic Federal University, 236041 Kaliningrad, Russia; 4 Amirkhanov Institute of Physics, Dagestan Federal Research Center, Russian Academy of Sciences, 367003 Makhachkala, Russia

**Keywords:** polyvinylidene fluoride, sensor, flexible electronics, piezoelectricity, structural properties

## Abstract

Currently, there is an ever-growing interest in carbon materials with increased deformation-strength, thermophysical parameters. Due to their unique physical and chemical properties, such materials have a wide range of applications in various industries. Many prospects for the use of polymer composite materials based on polyvinylidene fluoride (PVDF) for scientific and technical purposes explain the plethora of studies on their characteristics “structure-property”, processing, application and ecology which keep appearing. Building a broader conceptual picture of new generation polymeric materials is feasible with the use of innovative technologies; thus, achieving a high level of multidisciplinarity and integration of polymer science; its fundamental problems are formed, the solution of which determines a significant contribution to the natural-scientific picture of the modern world. This review provides explanation of PVDF advanced properties and potential applications of this polymer material in its various forms. More specifically, this paper will go over PVDF trademarks presently available on the market, provide thorough overview of the current and potential applications. Last but not least, this article will also delve into the processing and chemical properties of PVDF such as radiation carbonization, β-phase formation, etc.

## 1. Introduction

Polyvinylidene fluoride -(CH_2_-CF_2_)n- has a set of valuable properties [1,2]: a relatively high melting point, high mechanical strength even at high temperatures, chemical and radiation resistance, resistance to hydrolysis and ultraviolet radiation, good wear resistance, physiological inertness, very low thermal conductivity, and exceptional resistance to ignition. The β-phase of PVDF has ferroelectric and piezoelectric properties which is why it is especially interesting. Therefore, PVDF is widely used in electronics, acoustics, radio engineering, medicine, pharmaceuticals, production of components for the petrochemical, chemical, metallurgical, food, paper, textile and nuclear industries, as a structural and packaging material, in the manufacture of solar cells and piezoelectric elements. Scientific interest in PVDF is due to the possibility of synthesis on its surface by carbonization of various forms of carbon: structures with sp^2^—and sp-hybridization of valence electrons [3,4]. Radiation carbonization will be also discussed in this review as it is important aspect of PVDF processing. The surface of polymers such as PVDF can degrade under electron bombardment or X-ray radiation and which leads to the decrease of the fluorine concentration. This process is known as radiative carbonization, and it is one of the possible methods for obtaining carbine chains on the polymer surface of sufficient length [5]. Furthermore, radiation carbonization can lead to the appearance of new properties (chemical and physical) in the polymer which enhances its application potential. Additionally, the carbon–carbon which form upon the removal of HF can connect with each other thereby forming chain-like structures such as: polycumulenes (double carbon-carbon bonds) or polyene (alternating triple carbon-carbon bonds). [6] Carbon substances consisting of such one-dimensional structures are referred to as carbinoids and can be used in optoelectronics or in the solid-state for emission electronics, not to mention other chemical and physical applications. There is also a possibility that the carbonized layer of PVDF will have superior conductivity compared to its polymer base [7,8,9,10].

PVDF is a highly reactive white crystalline thermoplastic fluoropolymer, unlike other fluoropolymers, it has a low density of 1.78 g/cm^3^. PVDF exists in various conformational states, its macromolecules can be in the state: disordered amorphous and ordered crystalline. Being a semi-crystalline thermoplastic, PVDF exhibits chemical, thermal and mechanical properties in a wide temperature range. Molecular weight of PVDF is above 100,000 g/mol, melting point is of 171–180 °C, the crystallization temperature is of 141–151 °C, and a glass transition temperature is of −40 °C. It is soluble in dimethylsulfonic acid, dimethylacetamide, dimethylformamide and insoluble in ketones and esters. It has high mechanical strength, wear and weather resistance, resistance to ultraviolet and ionizing radiation, the action of mineral acids, with the exception of fuming sulfuric, alkalis, halogens and hydrocarbons. It is easily dyed in bright colors [1,2,3,4].

It is important to provide a summary of the literature on PVDF that has been published so far. Table 1 contains a list of various published review papers related to PVDF. Most of them focus on the applications of the polymer for specific purposes. This particular review besides containing complementary information also provides a more general assessment of PVDF properties, its application potential and processing methods.

The uniform distribution of fluorine and hydrogen atoms along the PVDF polymer chain facilitates the possibility of HF (hydrogen fluoride) removal both in the polymer chain itself with the formation of conjugated double bonds and an increase in thermal stability, and between adjacent polymer chains with the formation of cross-linked structures. During the pyrolysis of PVDF, C–C bonds can also be broken with the appearance of short-chain fragments of the polymer and free radicals as a result of disproportionation, which makes possible the chain process of degradation of a part of the polymer to a monomer [21]. PVDF is mechanically strong and flexible.

PVDF has been the dominant polymeric binder in the mass production of lithium-ion batteries (LIB) for decades [22,23]. PVDF belongs to the class of fluorinated polymers and is therefore characterized by a high melting point, chemical resistance to solvents and high electrochemical stability (up to 5 V). This polymer has a good binding capacity and, at the same time, swells to a limited extent in the electrolyte, facilitating the transfer of lithium ions to the surface of the active material [22,24]. Partially crystalline PVDF has several polymorphic modifications; however, it predominantly crystallizes with the formation of the α-phase, the formation rate of which is maximum. The degree of crystallinity of PVDF depends on the crystallization conditions and can reach 50–70% [25]. The high degree of crystallinity determines the minimum degree and low swelling rate of PVDF in the organic electrolyte, ensuring long-term and stable LIB operation. At the same time, the amorphous region in PVDF is a good matrix for the electrolyte, due to which lithium ions can pass through a thin layer of a swollen polymer binder [25].

It has a combination of valuable properties: a relatively high melting point, high mechanical strength, resistance to moderately aggressive media, good biocompatibility. One of the modifications (β-phase) of PVDF has ferroelectric and piezoelectric properties. The polymer is widely used for anti-corrosion coatings, manufacturing of acoustic devices, as a structural and packaging material. The unique combination of physicochemical characteristics allowed this polymer to be included in the so-called NASA list of materials promising for use in space [26].

The priority in obtaining high molecular weight PVDF, which is a technically useful product, belongs to ‘Du Pont’ company. The method patented by her is a water-suspension process under the action of such initiators as benzoyl peroxide, ammonium persulfate or molecular oxygen. Particular attention is paid to electroactive polymers capable of efficiently converting mechanical action into an electrical charge. A more promising representative is polyvinylidene fluoride (PVDF), which is used as sensors and transducers of acoustic signals. Unlike other polymers of fluorine-substituted ethylene, the electrical properties of PVDF do not allow them to be used as high-frequency insulation; however, the high values of the dielectric constant, the presence of ferroelectric and pyroelectric properties [27] make it promising to use the polymer and compositions based on it in acoustic and pyroelectric converters [28].

Polyvinylidene fluoride PVDF appeared on the world market in the early 1960s and was originally used for the manufacture of packaging films and protective coatings. The piezoelectric effect in PVDF films was first discovered by Japanese researchers in 1969 [29].

PVDF forms at least four distinct crystalline phases. The two most important are α, formed under normal crystallization conditions, and β, formed as a result of pressure crystallization or mechanical deformation of polymer films. The first is a thermodynamically stable structure and consists of chains with a trans-gauche conformation. In the latter case, the chains have a flat zigzag structure with fluorine on one side and hydrogen on the other. This polar structure is responsible for the piezoelectric and pyroelectric properties of PVDF [30].

The Japanese scientist Kawai noticed a strong piezoelectric effect in PVDF films, and also found that some plastics, such as polyvinyl fluoride (PVF) and polyvinylidine fluoride [31], have strong piezoelectric properties. It also turned out that these materials are also pyroelectrics. In 1975 Pioneer, Ltd. released the first loudspeakers and headphones based on PVDF [32]. In this case, the piezoelectric coefficient of thin films poled (placed in a strong electric field to create a total dipole moment) reached 6–7 PC/N: 10 times greater than that of any other polymer [29].

The main characteristics make this material very attractive for producing large-area PVDF films. The use of PVDF as coatings is made possible due to its thermal stability, resistance to creep at elevated temperatures and deformation under load, high degree of crystallinity, low permeability to gases and liquids, good resistance to mechanical damage—abrasion and cutting, corrosive effects, atmospheric—and fungus resistance, good dielectric properties. A distinctive feature of this PVDF fluoropolymer from other widely used ones is its ferroelectric properties (spontaneous polarization in the crystal).

PVDF is a semi-crystalline polymer with a degree of crystallization of 50%. PVDF films strongly absorb IR rays in the wavelength range of 7–20 µm. On the basis of PVDF films, sensors for the people movement are realized, as well as pyroelectric sensors for more complex devices, such as video cameras for night surveillance and laser copiers. Not so long ago, an IR matrix based on a PVDF film was presented, which makes it possible to identify fingerprints, using the pyroelectric effect inherent in polymers [33].

The widespread use of polyvinylidene fluoride is due to its consistent performance in certain high focus applications in products with particularly high requirements for ultraviolet radiation, microbiological damage, abrasion, scratch resistance, combined with relatively low cost.

The material is used in many electronic components, especially as a sheath material in cables used in voice and video transmission devices, as well as in alarm systems. PVDF does not burn well and emits little smoke, which is the main factor that allows it to be used in these areas.

New PVDF copolymers developed in recent years have found a wide range of applications in piezoelectric polymer sensors. Such copolymers are used at higher temperatures (135 °C), and new forms of sensors can be obtained from them: cylindrical and hemispherical. They can be used to produce sensors whose thickness exceeds the limits for devices based on PVDF films: for example, silicon sensors with an ultra-thick (200 A) coating and sonars with a cylinder whose wall thickness exceeds 1200 microns. Piezoelectric cables are also made from copolymers. Copolymer films can be used and stored at temperatures not exceeding 135 °C, and PVDF films are recommended for use at temperatures up to 100 °C.

The insolubility and electrical properties of the material are explained by the presence of different polarities of alternating CH_2_ and CF_2_ groups located along the polymer chain.

PVDF dissolves at room temperature in polar organic solvents and forms homogeneous mixtures with some carbonyl-containing polymers. It has good chemical and oxidative stability but is much more sensitive to organic and inorganic bases [30]. Due to the good combination of properties and processability, PVDF has become the second most used fluoropolymer (after PTFE).

Melt processable fluoropolymers are a class of high-performance engineering plastics used in many niche industries through traditional processes such as injection molding and extrusion. PVDF is the only melt-processable fluoropolymer that is produced from a single repeating structure (VF2). In most of these areas, PVDF is processed by extrusion and injection molding, where polymer specifications are designed for these processes so that molecular weight can control melt viscosity.

The good processability of PVDF from the melt facilitates the production of products from it by compression molding (bulk containers) and injection molding (sheets, coatings, plates and rods, films). Most often, PVDF is produced in the form of tubular products (tubes and fittings, pumps, valves), and ultrapure water is transported through PVDF pipelines. Manufacture of housings for quick couplings, locking sleeves, adapters, for a specific application and with the appropriate characteristics, are made from various polymeric materials: polyimide, polypropylene, synthetic materials (for example, PVDF). PVDF is used in the equipment of chemical, semiconductor industries (actuators, sensors and loudspeakers, plates and insulators for premium-class wire); as a binder in the production of cathodes and anodes for lithium-ion batteries (supercapacitors, battery separator in lithium-ion polymer systems); in the medical and defense industries, as well as in new areas for the manufacture of components for air and sea vessels and the interior of office equipment, ultrafiltration membranes and membranes for fuel cells.

## 2. PVDF Trademarks

Today, PVDF is marketed under many different brands: PVDF is sold under a va-riety of brand names including KF (Kureha, Düsseldorf, Germany), Hylar (Solvay, Brussels, Belgium), Kynar (Arkema, Budapest, Hungary) and Solef (Solvay).

Arkema is the world’s largest producer of PVDF. Its manufacturing facilities are located in the US, France and China. Arkema produces both homopolymer and copolymer grades under the Kynar^®^ and Kynar Flex^®^ brands. Kynar^®^ brand polyvinylidene fluoride (PVDF) began in 1965 for chemical handling applications and electrical wire insulation and sheath materials, and by now Kynar^®^ PVDF has become one of the materials of choice for many applications requiring high performance. Kynar Flex^®^ PVDF was commercialized in the 1980s as a material similar in performance to Kynar^®^, but with more versatility to meet the demands of new applications.

The new Kynar High Melt Strength (HMS) PVDF grades are molecular chain branched polymer resins that have high melt strength and resistance to sag during extrusion, making them candidates for future use in blow molding, thermoforming and film extrusion with bloat. This material provides an excellent balance of melt strength and elongation, high sag resistance at low viscosity, and high die swell. These improved properties are obtained by introducing a branch to form a long side chain.

Solvay Solexis now offers a growing selection of PVDF grades that are associated with new applications such as oil and gas, automotive, construction, electronics, chimney liners, lithium batteries, fuel cells, food and pharmaceuticals. In addition to Solef ^®^ and Hylar ^®^ PVDF resins, Solvay Solexis offers a wide range of other fluoropolymers that are also easily processed by injection, extrusion and all conventional processing methods:

- Halar ^®^ ECTFE (ethylene-chlorotrifluoroethylene copolymer),
- Hyflon ^®^ PFA (a copolymer of tetrafluoroethylene and perfluoroalkyl vinyl ether).
- Among the most important properties and advantages of PVDF Solef^®^ are the following:
- Excellent chemical resistance. The use of Solef^®^ resins, polymers of vinylidene fluoride, provides excellent corrosion and abrasion resistance when working with harsh chemicals.
- Excellent thermal stability.
- Fire resistance. Solef^®^ PVDF resins are rated UL-94 V-O.
- Purity Solef^®^ PVDF Resin is an extremely pure polymer free of stabilizers, plasticizers, lubricants and flame retardants.
- Increased abrasion resistance.

There are at least three varieties of PVDF on the market, differing from each other in the quantitative content of polyvinylidene fluoride and acrylic polymer: 50/50%, 70/30% and 80/20%. All of these varieties can be called PVDF. However, licensed PVDF is produced only under the well-known Kynar500 or Hylar5000 licenses and contains at least 70% polyvinylidene fluoride. 

Georg Fischer’s PVDF pipes, fittings and valves are a high-performance piping system with good impact resistance in all climatic conditions. Excellent chemical resistance, abrasion resistance up to the highest temperatures. Georg Fischer manufactures PVDF pipes, fittings and valves under the brand name SYGEF^®^. Georg Fischer’s standard PVDF products are labeled SYGEF, additionally cleaned and packaged in a special way, products for the transport of very pure water (UPW) are labeled SYGEF PLUS.

- Nominal pressure up to 16 bar.
- Temperature ranges from −20 °C to +140 °C.
- SYGEF^®^ Standard—A one-piece and reliable solution for the transport of water and chemicals, also at high temperatures.

SYGEF^®^ Plus—Double Packed High Purity (HP) piping system is widely applied in hot ultrapure water (HUPW) and microelectronics industries. Manufactured, cleaned and packaged in clean conditions up to ISO class (100), SYGEF Plus delivers superior leaching performance combined with high reliability and long product life.

## 3. Overview of Applications and Properties

### 3.1. Properties 

One of the most important tasks of modern thermophysics and molecular physics is to establish the relationship between thermophysical properties (TPP), in particular thermal conductivity, of polymeric materials with their structure at various levels of its organization and the nature of thermal motion. Knowledge of this relationship makes it possible to deeply and comprehensively analyze the mechanism of heat transfer in polymer composite (PC) materials, which will help accelerate the solution of the problem of obtaining polymer materials with predetermined TPP. In particular, due to its relative technological simplicity, efficiency, and economy, the physical method of introducing various fillers into the polymer has received wide recognition [34].

The defining advantages of PVDF, which allow it to take one of the leading positions in the world market in a number of industries, are:

(1) Heat resistance to temperature fluctuations from −40 to +140 °C. Among fluorine-containing polymers, polyvinylidene fluoride (PVDF) is one of the most durable. It has good ductility, impact strength, and flexibility. The properties of PVDF are described in sufficient detail in [27,28,35,36,37]. The structure of PVDF macromolecule chains has been studied by many researchers who have used various techniques for this purpose [28,37,38].

(2) Purity of the composition is due to the components of the main substance that satisfies the necessary performance characteristics: it does not contain UV and heat stabilizers, various lubricants, softeners and other additives [39].

(3) Eco-friendliness—completely non-toxic and fully recyclable. It does not emit toxic, chemically harmful fumes into the environment even when melted [40].

(4) Protection against microorganisms—the polymer has very low adhesion to microorganisms (growth of fungi, algae and microbial films). Validity of its use for the storage of medical and food liquids; filters (filtration housings), track membranes, membrane packages, separation equipment and separation elements are expediently used: microfiltration for wastewater treatment and processing; seawater desalination; separation of liquids for cleaning oil, natural gas [41].

(5) Fire-resistance PVDF is a barely flammable material. Melts slowly without emitting much smoke.

(6) Easy processing allows the production of products of various shapes: by extrusion, injection molding, compression molding and machining. When heated, PVDF loses its rigidity and becomes ductile, which makes it possible to process end products from this material. It is easy to perform various connections and assembly of structures (butt and socket welding, flange and threaded connections). Furthermore, the PVDF can be modified by various additives to tailor its chemical and physical properties [42].

(7) Reliability, optimal wear resistance, the required mechanical strength of parts during installation and in industrial installations are provided by materials from polyvinylidene fluoride. Products are resistant to mechanical damage, surface abrasion, ozone, moisture and corrosion [43].

(8) Durability of products—have high resistance to aging and can last more than 50 years without loss of physical and mechanical properties [44].

PVDF shows little to no deterioration in mechanical properties after many years of outdoor use, which is why it is often used for coating the exterior surfaces of buildings. PVDF also stands out for its mechanical properties among conventional crystalline polymers: it ranks second after polyoxymethylene in terms of tensile strength, flexural stress and compressive stress, stiffness and hardness, has the highest impact strength, high coefficient of curing (80–90% tsri thickness 100 microns) in the visible region of the electromagnetic spectrum [36].

(9) Low surface roughness—high flow rate (very smooth inner wall surface).

(10) Chemical resistance—the main indicator of resistance to most organic and inorganic chemically aggressive liquids (acids and bases, alcohols and petrochemical solvents) even at elevated temperatures.

### 3.2. Applications

- In the chemical/petrochemical industry—non-flammable, can be used in fire and explosion hazardous industries; filtration of aggressive media from particles of microimpurities; does not react with other compounds. Compatible with thermoplastic materials and has excellent inert resistance to concentrated inorganic acids even at high temperatures (the main material for storing concentrated nitric acid), aliphatic solvents, ionic and salt solutions, which is essential for the safety of roofing and wall cladding during long-term operation (up to forty years), excluding the effects of acetone, highly concentrated alkali and other solvents (strong bases, caustics, esters, amines, ketones).

In terms of resistance to UV rays and rays at 100 Mrad, PVDF is an exception [20] PVDF has very good weather resistance and high flexibility. Therefore, it can be used to make coatings that transmit sunlight, PVDF also has good chemical resistance; even at high temperatures (363 K), PVDF is not affected by inorganic acids, corrosive materials (halogens, oxidizing agents), weak bases and salts, aliphatic, aromatic and chlorinated solvents [34].

- In nuclear power engineering—handling of nuclear waste. PVDF is used as pipes, sheets and linings in high temperature, hot acidic, radiation environments due to PVDF’s resistance characteristics and upper temperature thresholds. As piping, PVDF is rated for temperatures up to 248°F (120 °C). Examples of uses for PVDF include nuclear reactor waste management, chemical synthesis and production (conventional sulfuric acid), airboxes, and boiler service pipes. When heated above 350 °C, polyvinylidene fluoride begins to decompose with the release of toxic substances containing fluorine, so it is important during the operation of the material not to expose it to critical temperatures, including fire [45].
- Aviation industry—seals, linings, gaskets and other products that provide flexibility and resistance to corrosion [46].
- Aerospace industry—in wiring coatings as a thermal barrier. It is also available as a cross-linked, closed-cell foam, which is increasingly being used in the aviation and aerospace industries, and as an exotic 3D printer filament [29].

Interestingly, PVDF is even used in the manufacture of some devices for the space industry—for example, some devices are made using this polymer, which are installed in a space probe that measures the density of cosmic dust outside the solar system.

- In electronics/radio engineering—PVDF electronic components are used as a dielectric, insulation of electrical wires (light weight, flexibility, high coefficient of resistance to heat transfer). The unique piezoelectric properties allow the material to respond to and influence electrical and/or magnetic fields. PVDF is in demand in the production of complex electrodes for lithium-ion batteries because it does not react with electrolytes and lithium [47].

PVDF film is used in radio-electronic devices to protect highly sensitive devices; for the manufacture of printed circuit boards; insulation of wires/cable sheaths; for obtaining electrets and pyroelements; in touch switches and microprocessors; solar collectors, etc. [48].

The piezoelectric properties of PVDF are used in the manufacture of tactile sensor arrays, low-cost strain gauges, and lightweight sound transducers. Piezoelectric PVDF panels are used in the Venetia Burney Student Dust Counter, a New Horizons space probe science instrument that measures dust density in the outer solar system.

- In electrical engineering—the resistance of PVDF insulation to cutting allows it to be used in wires for panels of computing devices, for aviation instrumentation and other types of electronic equipment. As a winding and insulating material, as a protective coating for special overhead cables, electrical equipment (motor windings).

Sensing elements based on piezoceramic materials have been used in the creation of hydroacoustic antennas since the 1940s to the present due to their high sensitivity and electrical capacitance. It is advisable to create electroacoustic transducers based on PVDF films, which, in addition to improving the weight and size characteristics, will reduce the susceptibility of antennas to hydrodynamic interference without the use of additional rigid structural elements, will allow designing antennas using flexible receiving elements of a large area [49].

PVDF membranes can be used as separators in lithium-ion batteries because they have good chemical and heat resistance. Such membranes have good mechanical strength, sufficient pore size and interruption characteristics. The material can also be used to obtain piezoelectric sensors and laser beam profile sensors in modern applications [15,16,17,18].

- In construction—as erosion-resistant, protective anti-corrosion and weather-resistant coatings (films and enamels). Applicable to the material—all types of welding non-contact infrared and seamless, except for chemical (adhesive bonding). PVDF material is commonly used in the manufacture of various parts and equipment for pumping stations and pipelines suitable for pumping ultrapure water and chemicals (acids, gases, organics). The PVDF polymer coating is good for responsible outdoor use (during the construction of industrial facilities; chemical production facilities; cladding of the facades of buildings located in the industrial zone, where the walls must be frequently washed not only with water, but also with disinfectant solutions), reliably withstands heating up to 120 degrees, and frost—up to 60 [50].

Filled PVDF film—veneer material in construction; the coating is applied to gas ducts, chemical cabinets and boxes, facade fixtures, composite panels, roofs. Sandwich panels coated with PVDF can be used for buildings with specific operating conditions in areas with an aggressive climate (high resistance to sunlight, frost resistance, high salt vapors (cowsheds, pigsties, poultry houses) [51,52].

- In architectural structures—which are subject to increased aesthetic requirements for walls and architectural and decorative details for the decoration of facades (to maintain strength, color and gloss longer) and as an anti-vandal coating (unauthorized inscriptions).

The main application of PVDF is architectural coating (Iezzi 1997). PVDF dispersions are applied to metals such as steel or aluminum to provide a decorative and weather resistant coating for commercial and residential buildings. It is used for electrical insulation, despite its higher permittivity, and in the chemical industry [30].

- In the automotive industry—PVDF-based laminating multilayer films are used for decorating external surfaces. Multilayer films based on PVDF are versatile materials that allow you to create both transparent coatings and coatings of various colors, metallic colors, mother-of-pearl, imitate the texture of wood, leather, and apply geometric patterns. Parts that can be produced with these PVDF-based films include taillight surrounds, side mirror housings, front grilles, bumpers, door handles, body side moldings, and more. Decoration during molding allows obtaining surfaces with better color and gloss retention parameters and exceptional resistance to chemical pollution [53].
- In pharmaceutical industry—PVDF materials provide special cleanliness and sterility of rooms—premises, and due to excellent deformation characteristics and heat resistance, they allow autoclaving; serve as packaging for medical instruments; Surgical sutures made of PVDF are resistant to chemicals (do not cause an allergic reaction) and have extremely high tensile strength [54].
- In biomedicine (biomedical research). Polyvinylidene fluoride proved to be an absolutely ideal and stable material (minimal tissue reaction, no capillarity and wicking, perfect antithrombogenic effect) for use in gynecology (obstetrics); general surgery (orthopedics, cardiovascular, plastic and reconstructive) [55].

At present, polypropylene mesh endoprostheses are widely used in domestic herniology. However, given the significant experience in the use of these materials, it can be said with confidence that these endoprostheses are not ideal for hernioplasty [56,57]. Most of the known polypropylene endoprostheses after implantation in the abdominal wall tissue cause an inflammatory reaction leading to the formation of a rough scar [58], which contributes to the wrinkling of the endoprosthesis [58] and disruption of the biomechanical parameters of the abdominal wall.

A promising way to prevent these complications is the use of polymers in hernioplasty that differ from polypropylene in chemical and physicomechanical properties. One such polymer is polyvinylidene fluoride (PVDF), which is widely used in reconstructive surgery [59,60,61,62]. Studies show that PVDF, like polypropylene, has bioresistance, strength and resistance to infection, surpassing the latter in elasticity.

Another feature of the PVDF polymer is the possibility of coating its surface with a linear-chain carbon-carbine [63]. It is now known that carbine applied to polymeric materials can significantly increase the biocompatible properties of surgical suture materials [64].

The purpose of the study [64] is to experimentally substantiate the possibility of using mesh endoprostheses based on PVDF-K to improve the immediate and long-term results of hernioplasty. The experiments consisted in comparing the biocompatibility of Esfil polymeric endoprostheses widely used in herniology from polypropylene monofilaments and new experimental PVDF-K endoprostheses. All studied materials had the same fiber structure and thickness. To study biocompatibility, these materials were implanted in the tissues of the anterior abdominal wall of animals in the on-lay position and fixed with the same name suture material (polypropylene and PVDF-K monofilaments, respectively), size 3/0. Comparative analysis of qualitative and quantitative changes in the tissues of the anterior abdominal wall in series with implantation of Esfil material and PVDF-K material confirms the best biocompatible properties of the carbine-coated PVDF-based endoprosthesis. Early relief of inflammatory changes, early maturation of granulation tissue, lack of encapsulation of endoprosthesis elements in the presence of a single histotypical structure of the emerging scar, and relatively rapid stabilization of the tissue response to the implant can be the overall result of adequate physical and mechanical properties of the PVDF polymer and pronounced biocompatible properties of the coating from carbine. All this gives the right to assert that the use of mesh endoprostheses based on PVDF-K will improve the immediate and long-term results of hernioplasty.

PVDF is a non-absorbable monofilament synthetic surgical suture: well suited for cardiovascular surgery where resistance to cardiac movement is required, both in cases of prosthetic valves and vascular anastomoses. PVDF is recommended for applying removable cosmetic intradermal sutures, is approved for fixing various implants (hernioprostheses, vascular prostheses), is widely used in mammoplasty operations when applying a circular Benelli suture around the halo, and is used in plastic surgery and microsurgery.

PVDF, an inert material, has long been used as a suture material. It has a high degree of biocompatibility, similar to that of polypropylene or polyester. Due to the low incidence of inflammatory and fibrotic foreign body reactions and low shrinkage after implantation, PVDF has been shown in some studies to be a useful alternative to conventional materials such as polypropylene and polyester [65].

PVDF membranes can be used in other biomedical applications as part of a membrane filter device, often in the form of a syringe filter or wheel filter. The various properties of this material such as heat resistance, resistance to chemical corrosion and low protein binding make this material valuable in the biomedical sciences for drug preparation as a sterilizing filter and as a sample preparation filter for analytical methods such as high-performance liquid chromatography (HPLC), where small amounts of particulate matter can damage sensitive and expensive equipment.

- In microelectronics and photonics, it has now been demonstrated that ultrathin PVDF films can be obtained using spin coating [66]. The production of ultra-thin, pinhole-free, smooth PVDF films paves the way for integrating ferroelectric and piezoelectric properties into microelectronic devices. Moreover, thin and smooth PVDF is a worthy candidate for photonic applications [67] due to its chemical and environmental stability. The surface roughness and smoothness presented here is sufficient to prevent optical losses in planar PVDF waveguides. In addition, PVDF can be used to incorporate complex functional materials providing electro-optical effects.

Fashion designer and professor Ying Gao’s latest designs, titled “Can’t” and “Won’t”, are two spectacular dresses made from “super organza” material, cotton and polyvinylidene fluoride. The latter is a piezoactive fluoropolymer capable of changing dimensions and/or bending under the influence of electricity. The highlight of the project is the “eye-tracking” system integrated into clothing, which is well known to smartphone users. Thus, clothes can be “activated” by the interested look of a nearby person.

- In the food industry—used in repeated contact with food, as it complies with FDA requirements and is non-toxic at temperatures below the decomposition temperature [29]. Production of containers for drinks; low thermal conductivity, eliminates the need for additional thermal insulation.
- Other applications. The aforementioned list of properties is not exhaustive. PVDF and materials on its basis also find applications in such areas as: textile industry, in the metallurgical industry, in the pulp and paper industry, etc. Furthermore, the characteristics of the polymer allow it to be used in the production of sorption columns. Such a product is suitable for the extraction of rare earth and precious metals: gold, uranium, copper, vanadium and others. The unit has low thermal conductivity and low weight. It has a high efficiency, due to the wide throughput and smooth surface—sorbates and metal products will not accumulate inside. Additionally, PVDF is also used to obtain porous tips (balls) in the production of ballpoint pens [11,12,13,14,15,16,17,18].

## 4. Experimental Data on PVDF

This chapter contains a brief overview of studies conducted in order to study PVDF’s processability as well as its interesting properties characteristics.

### 4.1. Relaxation Transitions

**Figure 1:**
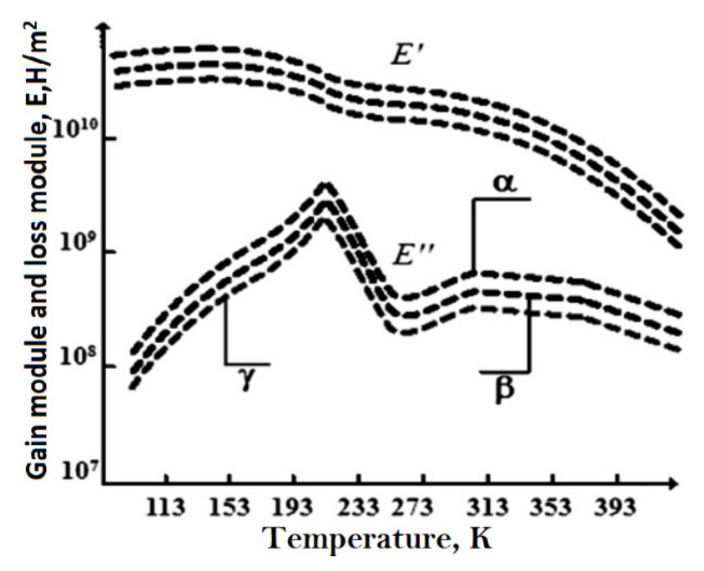
Temperature dependences of the dynamic mechanical characteristics of PVDF: storage modulus (E’) and loss modulus (E”) of isoth ermally crystallized and directionally crystallized samples of the α-phase of PVDF: 1—parallel; 2—perpendicular; 3—isothermally crystallized sample (spectra are numbered from top to bottom) [34].

Four main relaxation transitions in the PVDF phase were determined by dynamic mechanical spectroscopy [38]. In Figure 1, the temperature dependences of the dynamic Young’s modulus (E) determined along and perpendicular to the direction of crystallization of the sample obtained by isothermal crystallization [34] are given.

γ-relaxation, which begins at low temperatures, corresponds to the amorphous phase and limited movement of chains, and specific ones—to rotations of chains in the amorphous region, γ-relaxation is associated with glass transition, it corresponds to the micro-Brownian motion of segments [34,37,38] of the amorphous region. α-relaxation is associated with the movement of molecules, which changes the dipole direction only along the chain axis, and not perpendicular to it [38]. It is possible that the relaxation behavior of PVDF varies not only at different temperatures, but also depends on the methods used to study these properties.

### 4.2. Piezoelectric Behavior

The most important piezoelectric coefficient of PVDF is *d_33_*, also known as the compression mode. *d_31_* is another common piezoelectric coefficient otherwise called transverse mode, which is associated with the application of mechanical stress at right angles to the polarization axis [18]. There are also shearing strain coefficients *d_24_*, and *d_15_*, which are oftentimes omitted due to their low magnitude [68]. PVDF are reported to have a very high *d_33_* (~49.6 pm/V) [69] which can be measured by several methods: static, quasi-static and laser interferometer method [70]. These methods, however, have their disadvantages and limitations. Static and quasi static method produce too much of an electric charge by applying force to the samples which causes biaxial stress. Additionally, small thickness of the films entails complicated stress alignment [71]. Laser interferometer method on the other hand is quite sophisticated and requires excellent alignment of optical parts [72]. There is also a relatively new method proposed in [70]. The authors utilized a pneumatic pressure rig with a sole cavity for the measurement of PVDF piezoelectric coefficients. The method involves usage of highly pressurized nitrogen gas and the following monitoring of the induced charge variation. Thus, the *d_33_* value was accurately measured without any associated stress effects.

**Figure 2:**
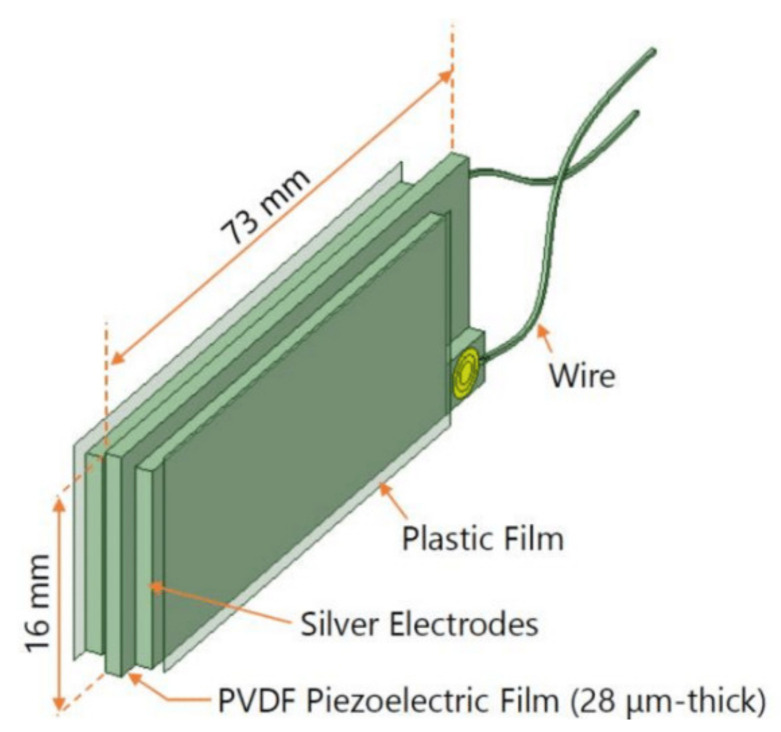
A schematic image of PVDF piezoelectric film (DT2-028K/L, Tokyo Sensor Co. Ltd.) which was utilized in the development of the respiration sensor [67].

Japanese scientists [67] from the Kagawa National Institute of Technology have developed a simple highly sensitive PVDF piezofilm sensor to monitor the respiratory status of public transport drivers, patients with artificial lung ventilation devices, as well as for screening tests of “apnea”—sudden cessation of breathing during dream. Figure 2 shows a PVDF piezoelectric film (DT2–028K/L, Tokyo Sensor Co. Ltd., Tokyo, Japan) used to develop a respiration sensor, which is 28 µm thick and has a surface area of 16 mm × 73 mm. Both surfaces of the piezo film are covered with silver electrodes, which are physically protected and insulated with a plastic film. Piezoelectric film generates electrical signals proportional to mechanical stress or strain, making it suitable for breath sensors due to its high sensitivity. The piezo film generates very large signals, even with minimal movement. Breath sensors based on PVDF piezofilms have already been developed and put into practice.

In [73], the piezoelectricity was studied using optical imaging and piezoresponse force microscopy (PFM) based on a flexible piezoelectric poly(vinylidene fluoride) (PVDF) film and the surface morphology of PVDF was analyzed. The authors, using theoretical modeling, investigated the interference of signals between adjacent matrices. The results indicate a reduction in interference as the pressure decreases at a rate of 0.028 mV/kPa, which depends significantly on the size of the electrode and becomes negligible at a pressure level of less than 178 kPa. These studies indicate that the electromechanical properties of the PVDF film sensor are characterized by both good piezoelectricity and excellent output characteristics and ultra-high signal-to-noise ratio.

**Figure 3:**
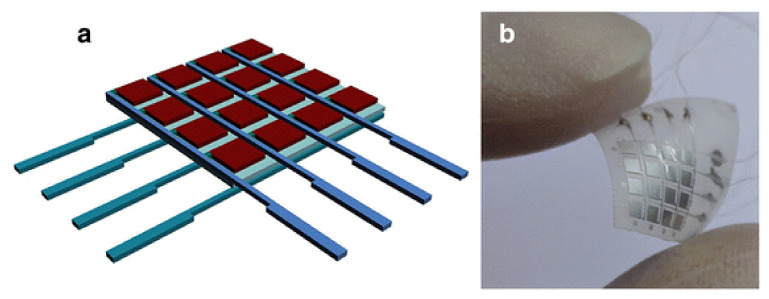
(**a**)—schematic diagram of the sensors matrix; (**b**)—physical picture of the end device [73].

The sensor matrix proposed by the authors has a sandwich structure based on a thin PVDF film with a thickness of ~50 µm (Jinzhou Kexin Inc., Jinzhou, China). The PVDF films was coated on both sides by aluminum electrode arrays 20 μm thick. Figure 3a shows a schematic diagram of the sensor and Figure 3b shows the real physical picture of the device and demonstrates its flexibility. The sensor has 16 blocks of micro-capacitors; every 4 blocks have one connection wire to keep the number of electrode wires to a minimum.

**Figure 4:**
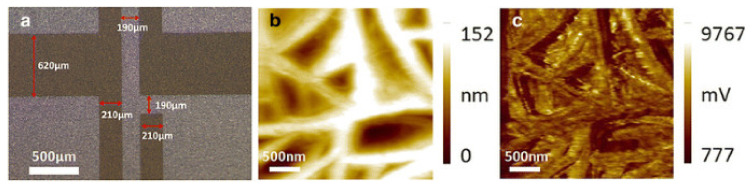
(**a**)—surface morphology of the proposed sensor after etching technology; (**b**)—Surface morphology and (**c**) PFM phase images of the PVDF sensor film [73].

Unlike piezoceramic transducers, sensors based on piezoelectric films have wider dynamic and frequency ranges. The wide frequency band (practically from 0 to 2 GHz) and low-quality factor can be explained by the softness inherent in polymers. Figure 4a shows the sensor surface morphology after Al etching, checked with an optical microscope. The rather bright and dark contrast suggests a clear interface between the PVDF and the etched aluminum electrodes.

Figure 4 shows the surface morphology and phase signal of the pressure sensor PVDF film. The surface of PVDF is stated to be smooth with a fabric structure. The phase image of the PFM measurement in Figure 4c shows a strong piezoelectric domain response which is in a good consistency with the surface structure observed in Figure 4b.

**Figure 5:**
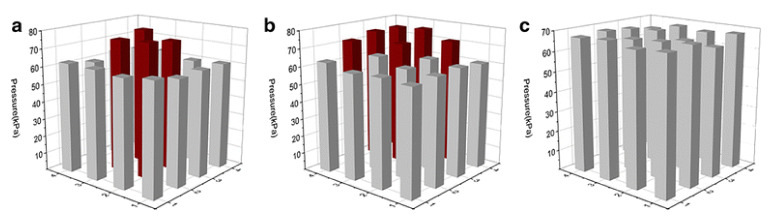
The state of pressure and distribution of movement of the thumb, characterized by the proposed sensor: (**a**) shiatsu, (**b**) kneading and (**c**) rubbing [73].

A sensor was utilized to measure the pressure state and its distribution caused by a finger of the human hand for a simple practical application. It is known that complex finger movement has some basic variations such as shiatsu, kneading, rubbing, etc. [74]. The three most commonly used movements, including shiatsu, kneading, and rubbing, were chosen to investigate the state of pressure and the finger surface area distribution. Figure 5 shows a diagram of the pressure distribution of the thumb, detected by the sensor, during three finger movements, correspondingly. In Figure 4a, it is apparent that the pressure of 76 kPa was focused at the center of the thumb during the shiatsu movement, which is differs quite strongly from the kneading and rubbing observed in Figure 5b,c, respectively. Figure 5b indicates that the pressure at the thumb front is higher than at other parts of the thumb during the kneading motion, whereas the pressure of the thumb is relatively uniform (about 68 kPa) when it comes to the rubbing motion, as shown in Figure 4c. The observed distribution of pressure in the finger bear some resemblance to the previous reports in clinical observations [75,76]. According to measurements, a load cell on the basis of flexible PVDF ferroelectric film demonstrated good sensitivity for characterizing complex finger movements. It is expected that the proposed sensor will be superior in terms of studying the human finger motion behavior, and it would also be useful for the development of a robot to replace human fingers in the future [74].

The author of [77] studied and obtained the properties of oriented and microporous PVDF films with piezoactive properties, as well as the development of composite materials based on microporous PVDF films with layers of an electrically conductive polymer (polypyrrole).

### 4.3. β-. Phase Formation

**Figure 6:**
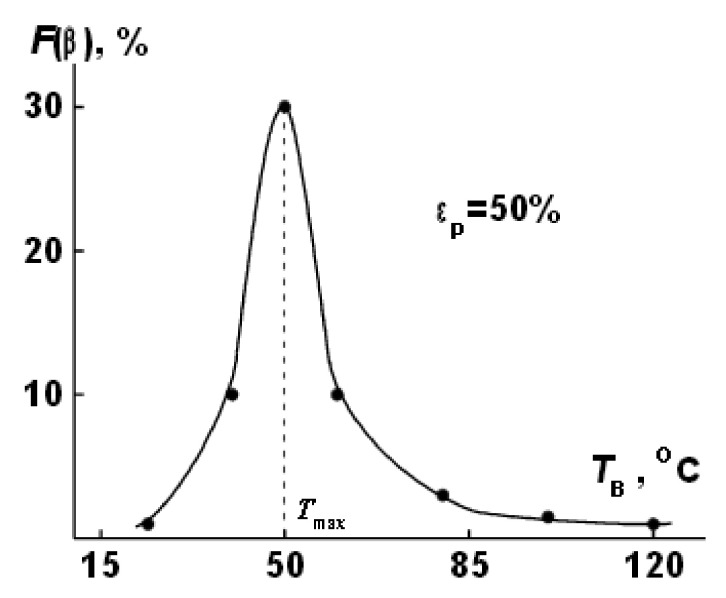
Dependence of the fraction of crystallites of the β-phase on the stretching temperature at a stretching ratio of 50% [77].

According to the author of [77], one of the most productive methods for the formation of β-form crystallites in PVDF today is the orientational stretching of the film (extrusion). One of the most important parameters in orientational stretching is the process temperature; it was found [77] that the best performance is achieved at a stretching temperature of 50 °C (Figure 6).

**Figure 7:**
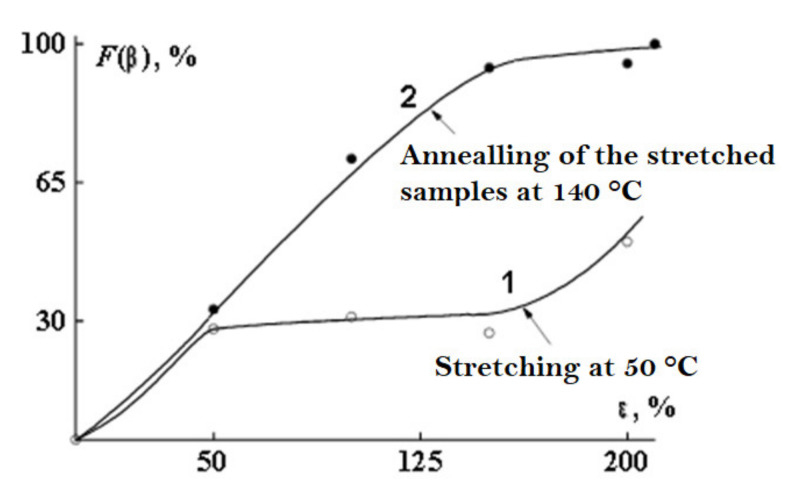
Dependence of the fraction of β-phase crystallites on the degree of stretching for PVDF films stretched at 50 °C (1) and for the same samples after annealing at 140 °C (2) [77].

After stretching, the film is annealed at 140 °C. At the stage of sample annealing, the crystal structure is improved (Figure 7).

As a result of annealing, the sample acquires rigid elastic properties and the ability of large reversible deformations with a high modulus of elasticity. It should be noted that, in a real sample, the formation of a porous structure is accompanied by the processes of orientation, rupture of passing chains, and destruction of crystallites. At the final stage—thermal fixation—there is a relaxation of internal stresses accumulated in the process of stretching. As a result, the formed structure becomes unstressed, and the dimensions of the cooled porous film do not change with time. It was found that isometric annealing of oriented polyvinylidene fluoride films leads to a significant increase in the content of p-shaped crystallites and an increase in the degree of crystallinity [77].

Special scientific interest in PVDF is caused by the possibility of synthesis on its surface by carbonization of various forms of carbon.

Based on PVDF, a thermionic cathode with a record low work function was obtained [78]. There are works on the synthesis of molecular magnets based on partially carbonized PVDF [79].

Chemical dehydrohalogenation of halogen-containing polymers (HCPs) is one of the most convenient and accessible methods for the synthesis of one-dimensional and quasi-one-dimensional carbon structures [80]. Among HCPs, PVDF is the most promising initial polymer for the production of articles from carbine due to its better solubility in the components of dehydrofluorinating mixtures [81]. A well-known method for smoothly changing the phase composition of PVDF composite films is their uniaxial mechanical stretching. An increase in the ratio of the final and -initial film sizes in the direction of stretching—the elongation factor—increases the content of the ferroelectric β-phase [82,83] and also promotes amorphization of the polymer substance in partially crystalline films [84]. To identify the molecular composition of carbinoids, to study its changes depending on the conditions and duration of carbonization, IR spectroscopy has been successfully used for a long time [85].

**Figure 8:**
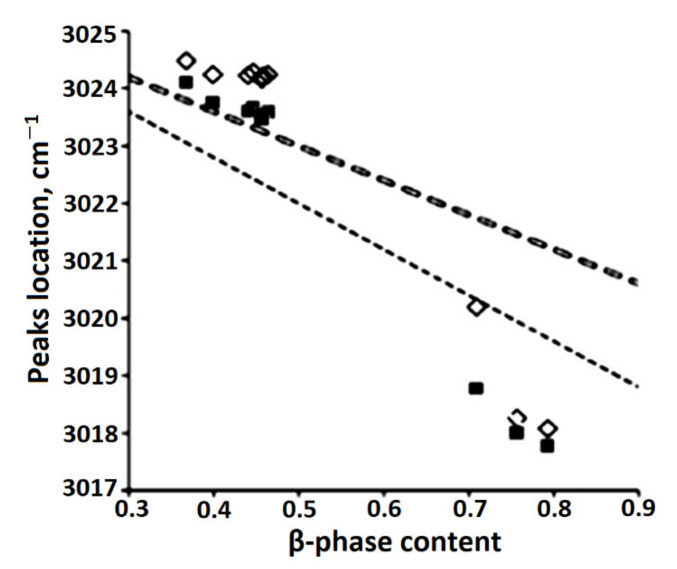
Dependence of the frequency position of peaks 1 and 2 on the concentration of the β-phase in the model and experimental IR spectra [26].

**Figure 9:**
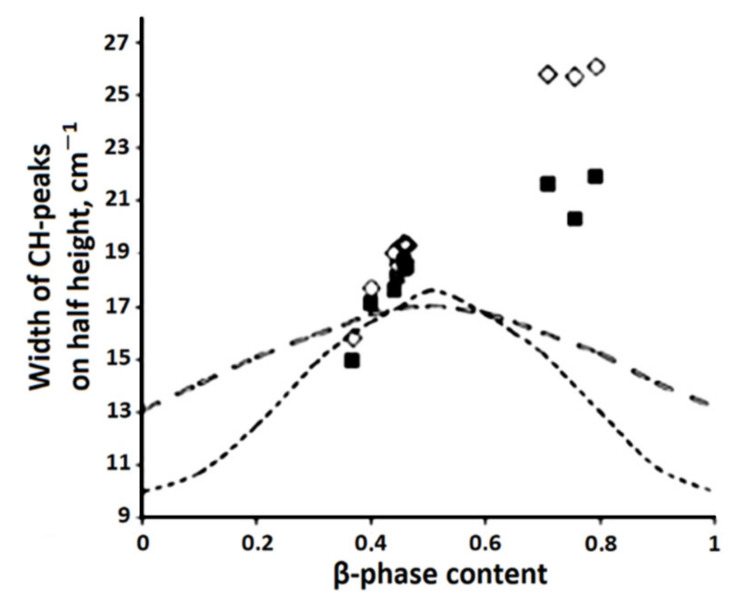
Change in the widths of CH peaks 1 and 2 depending on the concentration of the β phase in the model and experimental IR spectra. The designations are the same as in Figure 8 [26].

**Figure 10:**
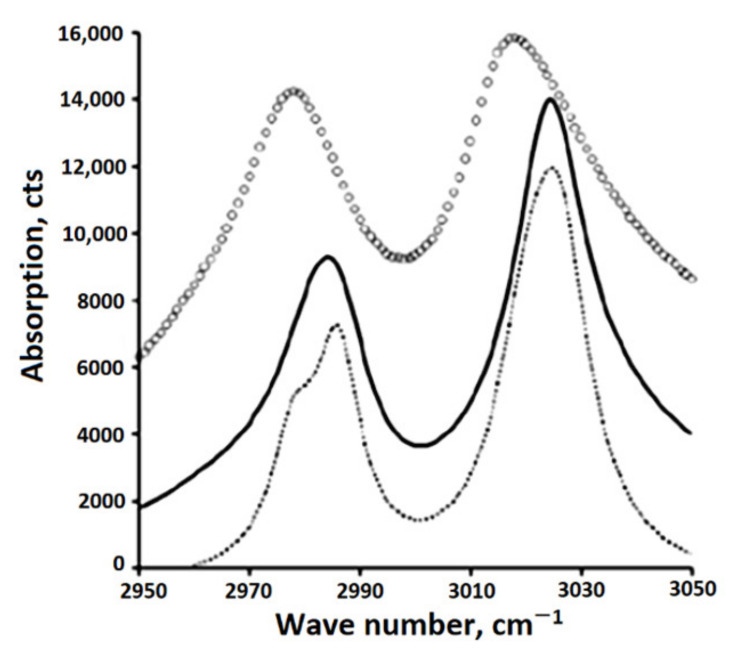
CH peaks of IR absorption of the experimental spectra of the maximally stretched (empty circles), initial (solid line) PVDF films and model simulation of a fragment of the spectrum of the initial film (dotted line). [26].

The authors of [86] synthesized a model of the CH absorption band, which makes it possible to measure the frequency position and width of the peaks with a change in the concentration of the β-phase in the sample. At the same time, a correlation was found between the parameters of the absorption band of CH bonds in the IR spectra of uniaxially stretched PVDF samples and changes in the phase composition of the PVDF film (Figure 8 and Figure 9. Designations ■ and ◊ correspond to the positions of peaks 1, 2). The parameters of the curves were chosen so that their sum (dashed curve, Figure 10) best described the CH absorption region in the spectrum of the unstretched film (solid curve).

The model developed under the assumption of an additive contribution of crystalline phases to IR absorption is inconsistent with the experimental data. One of the reasons for the discrepancy between the model and experiment may be the still unexplored effect of the amorphous component on the shape of CH peaks [26].

One of the directions for the synthesis of chain carbon nanostructures is the carbonization of polymers whose chains have a carbon skeleton, for example, polyvinylidene fluoride (PVDF). PVDF itself has a number of useful properties due to which it is widely used in membrane technologies [29], electronics, medicine, acoustics, etc. [6,86]. Its molecules are carbon chains, to each atom of which two atoms of fluorine and hydrogen are alternately attached. There are three main types of chain conformation: α, β, and γ [6].

Two main methods of PVDF carbonization are known: radiation (irradiation with quanta and bombardment with microparticles of various energies) [82,83,84,85,86,87,88,89] and chemical [6,90,91,92,93,94,95,96,97,98,99].

The most productive method for the deep carbonization of PVDF, which makes it possible to modify a sufficiently large amount of polymer without creating special conditions, is chemical carbonization [81,84,96,97,98,99].

Research on the properties of chain carbon and development of methods to improve the synthesis of carbon-based nanomaterials is of fundamental interest.

In their work [100], using IR spectroscopy, the authors revealed changes in the molecular composition of polyvinylidene fluoride as a result of chemical dehydrofluorination and subsequent storage at normal and reduced air pressure (a sample of PVDF grade F-2ME with a thickness of 20 μm). During chemical dehydrofluorination of polyvinylidene fluoride, fluorine-substituted polyene fragments are formed, as well as conjugated double and triple carbon-carbon bonds. Attachment to the carbon chain of hydroxyl groups contained in water, components of the dehydrofluorinating mixture and atmospheric air prevents the formation of conjugated carbon-carbon bonds. Drying under reduced pressure of samples dehydrofluorinated in a liquid medium promotes the formation of more extended chain fragments in which carbon atoms are interconnected by multiple bonds. The observed increase in the IR absorption of triple carbon/carbon bonds in the region of 2050–2100 cm^−1^ most clearly demonstrates the appearance of carbine-like atomic ordering of the polyyne type.

In works [101,102] on the study of the effect of heat treatment at 250 °C and above on the products of chemical carbonization of PVDF, it was possible to reveal the previously unknown effect of an abrupt multiple amplification of the EPR (electron paramagnetic resonance) signal with a change in its parameters—the width and position of the absorption line, which indicates the formation of a new paramagnetic carbon substance. Such controlled paramagnetic activity makes it possible to further expand the proposed area of practical application of the products of partial carbonization of PVDF. An amazing temperature dependence of the absorption EPR of chemically dehydrofluorinated samples was revealed, which indicates the presence of an activation contribution to the paramagnetic susceptibility [103].

Processing of films of PVDF grade F-2ME and products of their chemical carbonization by methods of synchronous thermal, gravimetric and mass spectrometric analysis revealed significant differences in the nature of fluorine desorption and changes in the masses of film samples of the original and partially chemically dehydrofluorinated PVDF during high-temperature (up to 600 and 800 °C) heat treatment in an inert atmosphere, leading to charring of the films and a very similar final state of their molecular structure [100,101]. During the chemical carbonization of the film, oxygen-containing groups are formed, which decompose at 430–650 °C. Chemical dehydrofluorination of PVDF leads to the formation of a carbon-enriched layer containing one-dimensional fragments on the PVDF surface. The addition of OH groups to the carbon skeleton prevents the formation of extended fragments dominated by multiple carbon-carbon bonds. In the early stages of dehydrofluorination (up to 3 h), the loss of methylene groups can be compensated by the addition of hydroxyl groups to the carbon skeleton. With an increase in the duration of dehydrofluorination, the number of multiple carbon–carbon bonds increases, as a result of which the possibility of such attachment is limited.

This leads to a slowdown in the rate of increase in the number of OH groups. Temperature treatment of a PVDF film chemically dehydrofluorinated for 15 h can significantly reduce the content of OH groups in it [32].

Previously [79,104,105], it was found that the aging of a partially chemically dehydrofluorinated PVDF film changes the EPR signal.

### 4.4. Chemical Carbonization

**Figure 11:**
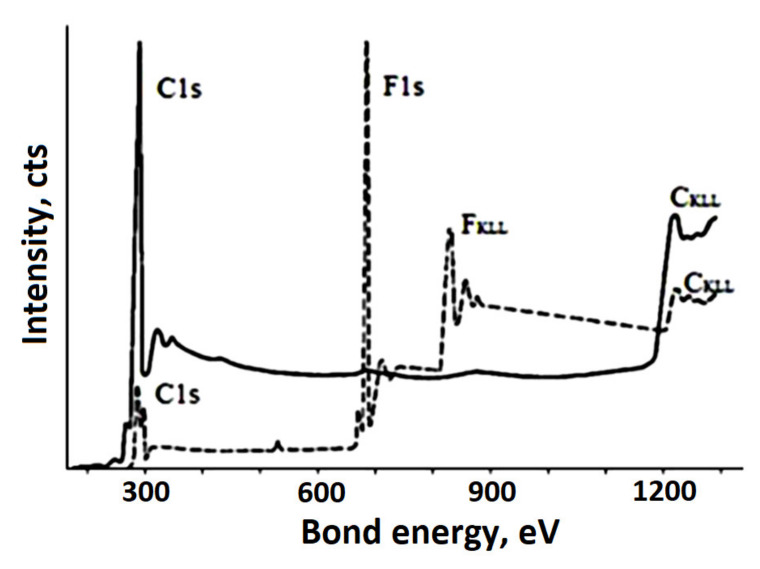
Panoramic photoelectron spectra of the original and dehydrofluorinated for 1 h at ~20 °C PVDF film (dashed and solid lines, respectively) [106].

In [106], an efficient dehydrofluorinating mixture for PVDF was proposed. The mixture consists of a saturated (20 wt.%) solution of KOH in ethanol and acetone in a volume ratio of 1:9, respectively. The authors consider PVDF as the most promising starting material for the synthesis of carbine due to its better solubility, although the dehydrohalogenation reaction in it proceeds more slowly than in other halogen-containing polymers due to the highest halogen–carbon bond energy in the series C–F > C–Cl > C–Br [107]. XPS spectra of the corresponding samples are given in Figure 11.

**Figure 12:**
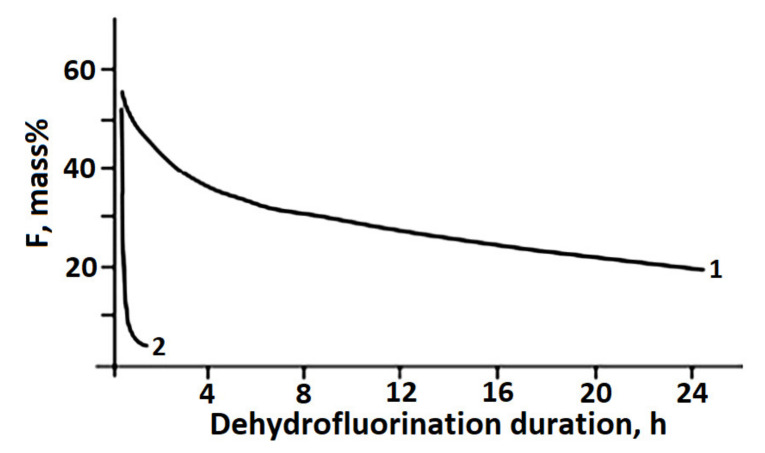
Dependence of the residual fluorine content in PVDF films on the DHF time during the reaction in an argon flow at (1) 22 and (2) 68 °C [108].

It was noted in [108] that the treatment of PVDF films with aqueous solutions of alkalis in the presence of ammonium and phosphonium halides at 70–90 °C for 24 h led to the formation of only fluorine-substituted polyene structures. With a similar chemical treatment of PVDF powders for 5–24 h, an insignificant amount of triple carbon–carbon bonds were detected. If, however, a mixture of a saturated solution of KOH in ethanol with tetrahydrofuran is used for dehydrofluorination of PVDF, then IR spectroscopic analysis demonstrates the appearance of double and triple carbon–carbon bonds. Figure 12 demonstrates how the HF content changed with time during dehydrofluorination reaction.

### 4.5. Radiation Carbonization

One of the methods for dehydrofluorination of PVDF to obtain chain carbon structures on its basis is radiation carbonization. For many polymers, the phenomenon of radiation degradation is observed when exposed to radiation and flows of particles of various nature. Studies of this phenomenon for polyvinylidene fluoride were carried out in [1,2,3]. It was shown in [2,3] that the radiation degradation of PVDF results in its carbonization due to dehydrofluorination. In radiation carbonization of polyvinylidene fluoride (PVDF), synthesized by exposure to AlKα photons and argon ions. The data obtained indicate a significant effect of the method of carbonizing treatment on the nature of the ordering of carbon atoms in the modified nanoscale layer of the polymer surface [109]. In this work, PVDF films of the Kynar brand (type 720, thickness 50 μm) produced by Atofina (France) by blow extrusion were subjected to radiation carbonization. The measurements showed that both methods of exposure (Al Kα photons and Ar+ ions) cause defluorination of the surface of the studied films; however, the rate of defluorination in the second case is much higher.

**Figure 13:**
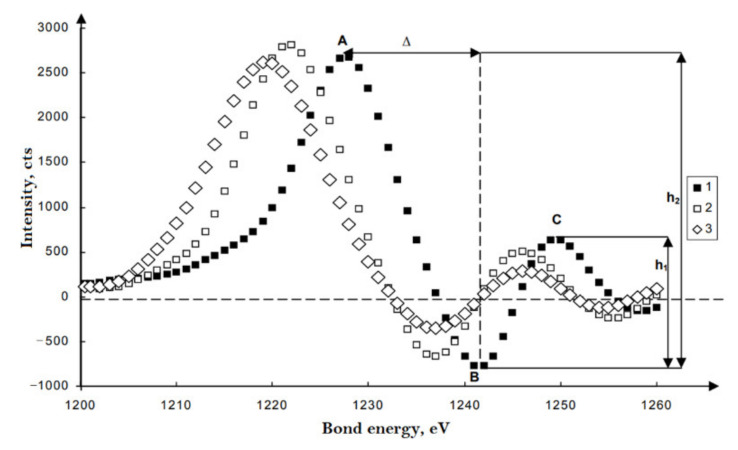
Derivatives of the Auger spectra of PVDF and its radiation carbonization products: h1 and h2 are, respectively, the intensities of singularities C and A with respect to the minimum of B; (∆) is the Galuska criterion (the difference between the energy positions of the minimum B and maximum A) [109]. (■)—original PVDF; (□)—PVDF subjected to X-ray carbonization at maximum exposure; (¯)—PVDF carbonized with ions at the maximum dose.

In Figure 13 the first derivatives of the smoothed C KVV spectra of the initial PVDF (series of dots ■) and products of its deep dehydrofluorination with photons and ions (series of dots (□) and (◊), respectively) are presented. All curves contain three dominant features A, B, and C, the energy positions and relative intensities of which differ markedly in the spectra of different samples. The results obtained demonstrate that the shape of the electron emission spectra of the carbonized layer of the film is significantly different for the cases of Al Kα irradiation with photons and Ar^+^ ions. In the near-surface nanolayers of films carbonized with ions, carbon structures with sp2 hybridization of valence electrons predominate. When irradiated with soft X-ray photons, the dominant type of hybridization in the carbonized layer differs from sp^2^. [109,110].

The mechanism of piezoelectricity in ferroelectric PVDF and its copolymers should take into account the structural-dynamic heterogeneity of flexible-chain crystallizing polymers. The presence of at least two phases (crystalline and amorphous) in the bulk of the film ensures the existence of three components of the macroscopically manifested piezoactivity [111].

The author of [111] considered the specific role of the condensed state in crystallizing polymers for the macroscopic piezoactivity in PVDF. PVDF and copolymers based on vinylidene fluoride (VDF), as representatives of the class of flexible-chain crystallizing polymers, are of interest to fundamental science due to the discovery of ferroelectricity in them [112].

For applied research, these compounds are also of interest due to the presence of piezo- and pyroelectricity in them [113,114]. Crystallizing polymers, as a class of condensed states of matter, have a number of specific properties. Among them is the coexistence of crystalline and amorphous phases in the bulk of the polymer. For PVDF, these phases at room temperature differ significantly in elastic and electrical characteristics [115].

**Figure 14:**
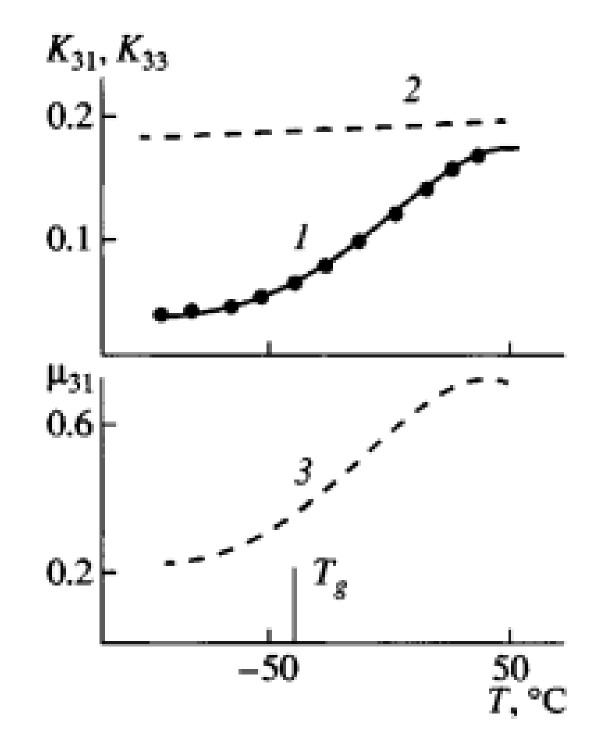
Temperature dependences of electromechanical coupling coefficients K31 (1) and K33 (2) [117] and Poisson’s ratio μ31 (3) for an oriented PVDF film [118]. Reprinted/adapted with permission from Ref. [XX]. Reprinted/adapted with permission from Ref. [118]. 2022, Elsevier”.

**Figure 15:**
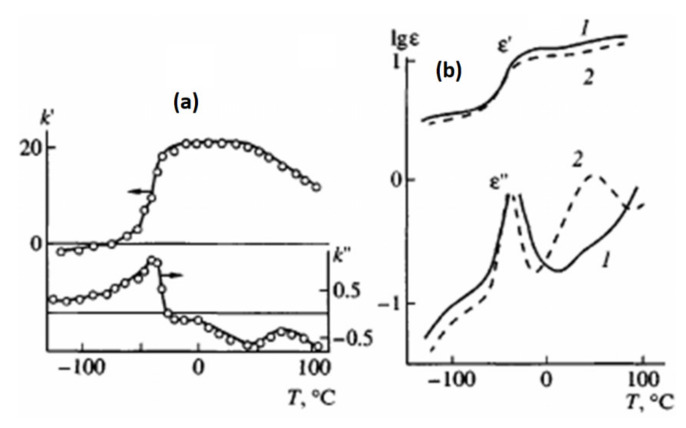
Temperature dependences of the components of the complex electrostriction constant (**a**) and permittivity (**b**) for oriented (1) and isotropic (2) PVDF films [119]. Reprinted/adapted with permission from Ref. [119]. 2022, AIP Publishing”.

Such heterogeneity is the reason for the complex mechanism of piezoactivity. Analysis shows that it has three components: the piezoelectric effect from crystals with a noncentrosymmetric lattice, electrostriction, and size effect [116]. For practical applications as various sensors [112,113], films of PVDF and its copolymers are textured most often by uniaxial drawing. On Figure 1. It can be seen that in a uniaxially stretched PVDF film, a decrease in temperature to the glass transition temperature is accompanied by a more than twofold decrease in the e31 piezoelectric constant. The dependence of the three parameters: ε, μ,k in the glass transition region for a single-stretched PVDF film is shown in Figure 14 and Figure 15; it can be seen here that all three parameters decrease upon transition to the glassy state.

#### 4.5.1. The Role of the Phase State for Piezoelectricity in Isotropic PVDF Films

In a number of cases, for sensors based on the materials under consideration, a requirement arises for the isotropy of characteristics in the film plane. Obtaining such sensors based on homopolymer films seems to be a difficult task in practice. The problem is that, under normal conditions of crystallization from a melt, a nonpolar α-phase is formed in PVDF [115], which is not piezoactive. There is a fundamental possibility of converting it into a polar modification αp or even into a ferroelectric α-phase due to polymorphic transformations in high-strength fields [115]. In practice, however, the fields required for this turn out to be higher than the breakdown ones, and the residual polarization cannot be obtained [120]. In this connection, the results of [121] are of interest, where the noted problem was solved by varying the crystallization conditions of isotropic PVDF films. It was noted that if crystallization from a melt proceeds at elevated pressure (more than 3 kbar), then the formation of a ferroelectric phase along with the nonpolar α modification is also possible [121]. By varying the pressure during crystallization, it was possible to obtain isotropic PVDF films with different ratios of a- and β-phases [111].

#### 4.5.2. Effect of Chemical Modification of PVDF Chains on the Characteristics of the Observed Piezoelectricity 

Another way to obtain isotropic films with ferroelectric crystals is the introduction of a comonomer of the tetrafluoroethylene (TFE) or trifluoroethylene (TrFE) type into the PVDF chain. It is known that these copolymers crystallize immediately in the polar β-phase even under normal conditions for preparing films from a melt [115].

One way to control the properties of films based on crystallizing polymers is their texturing. Therefore, the influence of such processes on the considered electromechanical properties is discussed below. With regard to this class of polymers, one of the most common methods of texturing is the uniaxial drawing of isotropic films. For PVDF, which is used for the manufacture of energy converters, such a procedure is necessary [111].

Thus, in [122,123], using the example of a number of PVDF films differing both in the synthesis conditions and in the thermal prehistory in the initial (isotropic) state, the influence of the drawing temperature Td and its multiplicity λ on the piezoactivity.

**Figure 16:**
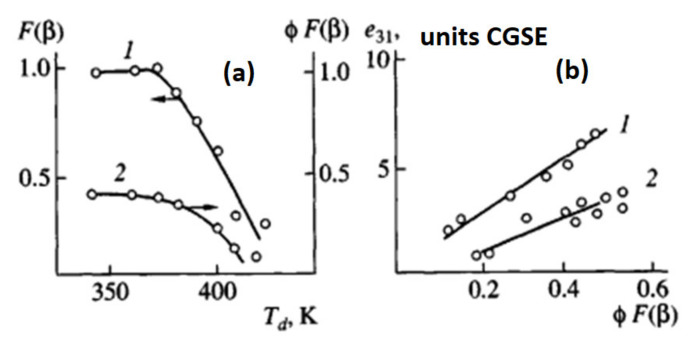
Change in the proportion of the β-phase F(β) in its mixture with the α-phase in the PVDF film (1) and the product of the degree of crystallinity φ by F(β) (2) as a function of Td with the drawing ratio λ = 4 (**a**), as well as the dependence of the piezoconstant e31 on φF(φ) after uniaxial drawing of the film (1) and its subsequent isometric annealing (2) (**b**) [120].

As can be seen from Figure 16, as the temperature of the uniaxial drawing of PVDF decreases, both the fraction of the polar β-phase F(β) and the product F(β) by the degree of crystallinity φ of the oriented film increase. From Figure 3b it follows that the dependences of the piezoconstant e31 are linear functions of the product φF(β). In [120], it is noted that on uniaxially oriented PVDF films, with an increase in the ferroelectric phase in them, an increase in the values of the polarization P_r_ was found.

To modify the properties of films of crystallizing polymers, their roller rolling is sometimes used. This method was also applied to PVDF films obtained by conventional uniaxial drawing [124].

In recent decades, there has been a search for various ways to improve the mechanical properties of polymer film materials. In particular, the technique of their formation from the dried gel has been developed. Its orientation can be carried out using solid phase co-extrusion. As applied to the polymers under consideration, this technique was used in [125,126].

The PVDF-based gel was prepared from a solution of PVDF in cyclohexanone prepared at 100 °C by cooling it; the solvent was removed from the gel by extraction with methanol. At an extrusion temperature of 160 °C, it was possible to obtain films with a stretching ratio of 8 [126] and even 10 [125]. Some physical characteristics of the obtained films are presented in Table 2.

Two main differences between the films under consideration follow from it. The film obtained from the gel has a 4 times higher modulus along the stretch axis than the film obtained from the melt. In addition, a film prepared from a gel has a significantly higher coefficient of electromechanical coupling in the transverse direction Kt (K33).

The mechanism of macroscopic piezoactivity in ferroelectric films based on PVDF should take into account the structural and dynamic heterogeneity of crystallizing polymers. The main contribution to the observed piezocontacts for the transverse effect is made by the size effect and electrostriction, for which the regions of the disordered phase are responsible. The noted effects should be interrelated, and the mechanism of their manifestation requires taking into account the occurrence of molecular rearrangements in the mobile phase of the polymer under the action of mechanical or electric fields. Progress in understanding the mechanisms of macroscopic piezoactivity (and, as a consequence, in its regulation) is largely determined by the general state of the problem of the structure of flexible-chain crystallizing polymers in isotropic and textured form. Knowledge of the fine details of the microstructure and dynamics of the regions of the amorphous phase may help in the future to create a rigorous theory of the phenomena under consideration [111].

The paper shows the possibility of obtaining films from modified polyvinylidene fluoride (PVDF-2M) by laser sintering. The effect of laser radiation with a wavelength of 10.6 μm on the polymer structure and the quality of film sintering was studied [127]. Before laser processing, the polymer was pressed on a PgPr manual hydraulic press at a pressure of 50 kgf/cm^2^ at room temperature: the dimensions of the pressed layer were 30 × 60 mm^2^ and the thickness was 0.8 mm. The polymer film up to 250 µm thick obtained as a result of laser treatment of the compact surface was mechanically separated from the green part. The authors of [127] found that the phase composition of the films practically does not change compared to the initial material, however, partial crystallization is observed when certain values of the power density and exposure time are reached.

In [128], the authors studied the influence of binder morphology (PVDF) on the cycling of a negative electrode based on a Sn/SnSb composite. It was found that in the case of dissolution of PVDF in a thermodynamically good solvent (NMP), the binder is evenly distributed inside the electrode, forming thin threads with a diameter of ⩽30 nm between active material particles. At the same time, when a thermodynamically poor solvent (decane) is used, the macromolecules form spherical particles ∼200–300 nm in diameter, nonuniformly distributed inside the composite electrode. At the same time, the resistance to cycling was much higher for electrodes of the second type with an uneven distribution of the binder. The authors explained this effect by the unequal binding force and different swelling of PVdF particles of different morphology, as well as differences in the porosity of the electrodes and the probable “buffer” effect of polymer particles. The results obtained in this work clearly show to what extent the behavior of a composite electrode during cycling is determined by its morphology; this is especially important to take into account for lithium alloys, which significantly change their volume during cycling.

The use of PVDF for the manufacture of graphite electrodes creates certain problems [129]. First, it is reported that PVDF covers up to 40–70% of the graphite surface, slowing down the penetration of Li^+^ ions into the depth of granules. Secondly, PVDF is predominantly adsorbed on the electrochemically active side faces of graphite particles (through which lithium intercalation/deintercalation occurs) and, due to its high viscosity, can aggregate into clusters, which additionally block the most reactive surface areas and reduce the charge transfer rate. Thirdly, weak adhesive bonds of PVDF with graphite are destroyed due to the expansion of its particles during lithiation and cannot be completely restored after delithiation due to the low flexibility of the polymer chain; this leads to a breakdown of electrical contacts between particles.

The disadvantages of PVDF, one of the most chemically stable binders, can be largely compensated by introducing the second and third copolymerization components into the macromolecules or by creating a mixture of polymers based on PVDF.

A convenient approach to modifying PVDF is the use of a mixture of polymers, which allows combining the advantages of materials. Recently, polypropylene carbonate (PPC) or a block copolymer of polyethylene and polyethylene oxide (PE-PEO) was added to PVDF. It has been shown that the addition of PPC reduces the degree of crystallinity of PVDF, increasing the interfacial adhesion in the electrode mass based on LiCoO2. PE-PEO macromolecules act as a surfactant for the conductive additive, which improves the distribution of the latter in the electrode mass. As a result, the specific capacitance and other characteristics of the obtained electrodes are better than when using individual PVDF. However, PPC and PE-PEO additions should not exceed 30 wt%, since this leads to a deterioration in the mechanical properties of the composite electrode.

The above examples show that the modification of polyvinylidene fluoride is a fairly effective way to improve the performance of electrodes. The introduction of functional groups into macromolecules makes it possible to change the nature of the interfacial interaction, varying it from van der Waals forces to hydrogen and even chemical bonding with the surface of the active material. At the same time, the modification does not completely eliminate the above disadvantages inherent in PVDF.

In this regard, a wide range of electrochemically stable non-fluorinated synthetic and natural polymers of various structures has been studied in recent years.

## 5. Future Prospects and Outlook 

As was demonstrated in this review paper, there is a great abundance of possible applications for PVDF and materials on its basis. Taking into account the plethora of PVDF research papers currently available and their ever-increasing number, we believe it is safe to assume that research on these attractive polymers will continue to thrive. Further modifications and combinations of PVDF with other materials to meet certain needs or criteria can be expected as well as more in-depth analysis of their properties.

A significant proportion of the current studies seems to be dedicated to the use of PVDF in lithium-ion batteries (LIB), as a separator [12]. This popularity is understandable considering a number of PVDF properties which make this material extremely suitable for this application: non-reactive nature, thermal stability, good mechanical strength, easy processability. We expect that the emphasis on this direction of development will remain in the nearest future.

In our opinion, another noteworthy potential application of PVDF that was mentioned in this paper is in biomedicine and surgery. For instance, currently there is no ideal material for anterior abdominal wall plastic surgery which makes the search for optimal polymers for surgical reconstruction of the anterior abdominal wall a substantial task. One of the promising materials in this regard is polyvinylidene fluoride (PVDF) which is widely used for the manufacture of suture material [130]. The characteristics reviewed by the authors [59,130,131,132] showed that PVDF monofilaments, unlike polypropylene ones, do not contain stabilizers and plasticizers, do not undergo hydrolysis under the action of tissue fluids, this explains their greater biocompatibility and resistance to the action of factors of the internal environment of the body during implantation. In terms of textile parameters, endoprostheses made of PVDF are more elastic than those made of polypropylene. According to the biological inertness of PVDF, mesh endoprostheses are close to porous film endoprostheses made of polytetrafluoroethylene, but they are significantly superior in resistance to infection and reliability of integration in tissues. To further reduce the tissue reaction to the endoprosthesis, a coating of linear-chain carbon-carbine (PVDF-K) was applied. In the process of forming a carbine coating on a PVDF endoprosthesis, there is a slight decrease in the strength characteristics of the material, but its elasticity practically does not change. The endoprosthesis PVDF has physical and chemical properties that ensure reliable prosthetics of abdominal wall tissues and is distinguished by good biological compatibility with human tissues. PVDF mesh implants are a promising alternative to the most common polypropylene endoprostheses in modern herniology [59,130,131,132]. In this regard, the investigation and improvement of PVDF to meet the needs of biomedical and surgical applications is a sensible direction for the future research.

## 6. Conclusions

This review was dedicated to PVDF applications, their trademarks, current position on the market of functional materials as well as to the study of the unique properties and processability. The list of properties that were discussed include (but not limited to): non-toxicity, fire-resistance, easy processing, heat resistance, resistance to aging, chemical resistance, low surface roughness, etc. These attractive characteristics naturally lead to a great variety of possible applications, such as: chemical, nuclear power engineering, aviation and aerospace, electronics and radio engineering, in architecture and automotive industries, in biomedical and pharmaceutical industries.

Among the most important aspects of the experimental section are: formation of the piezoelectric β-phase and radiation carbonization. β-phase of PVDF is of particular interest as it facilitates conversion of mechanical movements into electrical responses and vice versa. Radiation carbonization method is really important as it provides a tool to remove hydrogen fluoride (HF) in order to increase the chain length or alter the end properties.

We conclude this review by saying that, hopefully, the information provided in this review article is evidence enough to the great prospects of PVDF materials (as well as its copolymers and materials on its basis), and to the importance of continuation of research in this area. As was demonstrated, a great number of industries stand to benefit from the development and improvement of this material. A large amount of fundamental and applied research on the subject is a testament to that. Review papers, on the other hand, are also important as they systematize the information and facilitate consumption.

## Figures and Tables

**Table 1:** A list of published review papers on PVDF and short descriptions of their contents.

Title of the Paper	Content Description
(1) Advances and prospects of PVDF based polymer electrolytes [11].	A review summarizing recent advances on gel polymer electrolytes and all solid polymer electrolytes on the basis of PVDF.
(2) Lithium-ion battery separators based on electrospun PVDF: A review [12].	A review emphasizing the potential of electrospun PVDF in batteries, and how it improves their performance.
(3) Insights and perspectives on graphene-PVDF based nanocomposite materials for harvesting mechanical energy [13].	A review describing how graphene and its derivatives can enhance PVDF capabilities in piezoelectric energy harvesting.
(4) PVDF based ionogels: applications towards electrochemical devices and membrane separation processes [14].	A review going over advantages of PVDF as a matrix material for iongels based on polymers.
(5) Poly(vinylidene fluoride) (PVDF) membranes for fluid separation [15].	A review focuses on (but not limited to) the usage of PVDF membranes for water distillation.
(6) Flexible PVDF based piezoelectric nanogenerators [16].	A review summarizing latest information on PVDF used in nanogenerators.
(7) Progress in the production and modification of PVDF membranes [17].	A review highlighting various surface modifications in order to improve fouling resistance of PVDF membranes.
(8) A Brief Introduction and Current State of Polyvinylidene Fluoride as an Energy Harvester [18].	A review underlining the advantages of PVDF for the use as a nanogenerator
(9) Polyvinylidene fluoride: A multifunctional polymer in supercapacitor applications [19].	A review focusing on the potential of PVDF as well as its nanocomposites and copolymers in the supercapacitor industry
(10) Design strategy of poly(vinylidene fluoride) membranes for water treatment [20].	A review dedicated to understanding the science and engineering behind the membranes’ preparations based on PVDF.

**Table 2:** Characteristics of textured PVDF films obtained by solid-state gel extrusion (a) and uniaxial drawing with isometric annealing of a sample crystallized from the melt (b) [126].

Film	φ, %	*f_c_*	Δn	*l*_100, 200_, nm	*l*_001_, nm	L, nm	C_f_, GPa	K_t_
(a)	55	0.993	0.0346	8.6	6.4	13.2	8.3	0.24
(b)	33	0.986	0.0287	9.0	6.7	10.3	2.0	0.15

Note: l_110, 200_—transverse size of crystallites, l_001_—longitudinal size of crystallites, L—long period.

## Data Availability

The data presented in this study are available on request from the corresponding author.

## Abbreviations

The paper contains the following abbreviations:

PVDF	Polyvinylidene fluoride
LIB	lithium-ion batteries
NASA	the national aeronautics and space administration
PVF	polyvinyl fluoride
PTFE	Polytetrafluoroethylene
IR	infra-red
HMS	high melt strength
HP	high purity
TPP	thermophysical properties
PC	polymer composite
UV	ultraviolet
HPLC	high performance liquid chromatography
FDA	Food and Drug Administration
HCP	halogen-containing polymers
EPR	electron paramagnetic resonance
TFE	tetrafluoroethylene
TrFE	trifluoroethylene
NPM	N-Methyl-2-pyrrolidone
PPC	polypropylene carbonate
PE-PEO	polyethylene and polyethylene oxide

## References

[1] Morilova V.M. (2014). Study of the Carbonization of Polyvinylidene Fluoride by Emission and Absorption Spectroscopy. Master’s Thesis.

[2] Baskin Z.L., Shabalin D.A., Vyrazheikin E.S., Dedov S.A. (2008). Range, properties and application of fluoropolymers of the Kirovo-Chepetsk Chemical Plant. Russ. Chem. J..

[3] Voinkova I.V., Ginchitskii N.N., Gribov I.V., Klebanov I.I., Kuznetsov V.L., Moskvina N.A., Pesin L.A., Evsyukov S.E. (2005). A Model of Radiation-Induced Degradation of the Poly(Vinylidene Fluoride) Surface During XPS Measurements. Polym. Degrad. Stab..

[4] Joh H.-I., Ha H.Y. (2013). Properties and Formation Mechanisms of Branched Carbon Nanotubes from Polyvinylidene Fluoride Fibers. Carbon.

[5] Voinkova I.V., Pesin L.A., Volegov A.A., Evsyukov S.E., Gribov I.V., Kuznetsov V.L., Moskvina N.A. (2007). Depth distribution of the fluorine concentration during radiative carbonization of PVDF. J. Surf. Investig..

[6] Heimann R.B., Evsyukov S.E., Kavan L. (1999). Carbyne and Carbynoid Structures Dordrecht.

[7] Calcagno L., Musumeci P., Percolla R., Foti G. (1994). Calorimetric measurements of MeV ion irradiated polyvinylidene fluoride. Nucl. Inst. Methods Phys. Res. B.

[8] Oshima A., Ikeda S., Seguchi T., Tabata Y. (1997). Temperature effect on radiation induced reactions in ethylene and tetrafluoroethylene copolymer (ETFE). Radiat. Phys. Chem..

[9] Zhudi Z., Jin C., Xinfang C. (1994). Crystallite damage studies on irradiated poly(vinylidene fluoride). Radiat. Phys. Chem..

[10] Knápek A., Dallaev R., Burda D., Sobola D., Allaham M.M., Horáček M., Kaspar P., Matějka M., Mousa M.S. (2020). Field Emission Properties of Polymer Graphite Tips Prepared by Membrane Electrochemical Etching. Nanomaterials.

[11] Wu Y., Li Y., Wang Y., Liu Q., Chen Q., Chen M. (2022). Advances and prospects of PVDF based polymer electrolytes. J. Energy Chem..

[12] Bicy K., Gueye A.B., Rouxel D., Kalarikkal N., Thomas S. (2022). Lithium-ion battery separators based on electrospun PVDF: A review. Surfaces and Interfaces.

[13] Pusty M., Shirage P.M. (2022). Insights and perspectives on graphene-PVDF based nanocomposite materials for harvesting mechanical energy. J. Alloys Compd..

[14] Sahrash R., Siddiqa A., Razzaq H., Iqbal T., Qaisar S. (2018). PVDF based ionogels: Applications towards electrochemical devices and membrane separation processes. Heliyon.

[15] Ji J., Liu F., Hashim N.A., Abed M.M., Li K. (2015). Poly(vinylidene fluoride) (PVDF) membranes for fluid separation. React. Funct. Polym..

[16] Lu L., Ding W., Liu J., Yang B. (2020). Flexible PVDF based piezoelectric nanogenerators. Nano Energy.

[17] Liu F., Hashim N.A., Liu Y., Abed M.M., Li K. (2011). Progress in the production and modification of PVDF membranes. J. Membr. Sci..

[18] Papež N., Pisarenko T., Ščasnovič E., Sobola D., Ţălu Ş., Dallaev R., Částková K., Sedlák P. (2022). A Brief Introduction and Current State of Polyvinylidene Fluoride as an Energy Harvester. Coatings.

[19] Rajeevan S., John S., George S.C. (2021). Polyvinylidene fluoride: A multifunctional polymer in supercapacitor applications. J. Power Sources.

[20] Zou D., Lee Y.M. (2022). Design strategy of poly(vinylidene fluoride) membranes for water treatment. Prog. Polym. Sci..

[21] Kuznetsov E.V. (1977). Workshop on Chemistry and Physics of Polymers.

[22] Chen H., Ling M., Hencz L., Ling H.Y., Li G., Lin Z., Liu G., Zhang S. (2018). Exploring Chemical, Mechanical, and Electrical Functionalities of Binders for Advanced Energy-Storage Devices. Chem. Rev..

[23] Lestriez B. (2010). Functions of Polymers in Composite Electrodes of Lithium Ion Batteries. Comptes Rendus Chim..

[24] Chou S.L., Pan Y., Wang J.Z., Liu H.K., Dou S.X. (2014). Small Things Make a Big Difference: Binder Effects on the Performance of Li and Na Batteries. Phys. Chem. Chem. Phys..

[25] Nagai A., Yoshio M., Brodd R.J., Kozawa A. (2009). Applications of PvdfRelated Materials for LithiumIon Batteries. Lithium-Ion Batteries: Science and Technologies.

[26] Morilova V.M., Koryakova O.V., Evsyukov S.E., Pesin L.A. (2011). Influence of Uniaxial Stretching of Polyvinylidene Fluoride Films on the Shape and Position of CH Peaks in IR Spectra. Herald of ChelGU. https://cyberleninka.ru/article/n/vliyanie-odnoosnogo-rastyazheniya-plyonok-polivinilidenftorida-na-formu-i-polozhenie-sn-pikov-v-ik-spektrah.

[27] Tansel T. (2021). Effect of electric field assisted crystallisation of PVDF-TrFE and their functional properties. Sens. Actuators A Phys..

[28] Rakhmankulov A.A., Davlatov F.F. (2019). Research on the effect of dispersed graphite grade GMZ on the thermophysical properties and structure of polyvinylidene fluoride. Int. Sci. Tech. J..

[29] Kawai H. (1969). The Piezoelectricity of Poly(Vinilidene Fluoride). Jpn. J. Appl. Phis..

[30] Chu C.C. (2013). Biotextiles as Medical Implants.

[31] Seiler K., Simon W. (1992). Principles and mechanisms of ion-selective optodes. Sens. Actuators B Chem..

[32] Tang T.K., Liu S.S. (1991). Principles and Materials for Manufacturing Electrochemical Sensors in Chemical Sensor Technology.

[33] Zhivulin V.E. (2016). Synthesis and properties of paramagnetic layers on the surface of polyvinylidene fluoride. Master’s Thesis.

[34] Rakhmankulov A.A., Khaidarov T.Z. (2020). Peculiarities of thermal motion in polyvinylidene fluoride. Sci. Educ. Cult..

[35] Rakhmankulov A.A. (1986). Influence of dispersed fillers on the structure and thermal conductivity of unmodified and modified polyvinylidene fluoride. Master's Thesis.

[36] Kakutani M. (1970). Dielectric Absorption of Oriented Polivinildenftuoride. J. Polym. Sci. Part A-2 Polym. Phys..

[37] Harris G.R., Preston R.C., DeReggi A.S. (2000). Impact of piezoelectric PVDF on measurements, standards and regulations for medical ultrasound exposure. IEEE Trans. Ultrason. Ferroelectr. Freq. Control..

[38] Lovlnger A.J. (1981). Crystallization of the P Base of Polivlnilidenftuoride from the Melt. Polymer.

[39] Kaspar P., Sobola D., Částková K., Dallaev R., Šťastná E., Sedlák P., Knápek A., Trčka T., Holcman V. (2021). Case study of polyvinylidene fluoride doping by carbon nanotubes. Materials.

[40] Kaspar P., Sobola D., Částková K., Knápek A., Burda D., Orudzhev F., Dallaev R., Tofel P., Trčka T., Grmela L. (2020). Characterization of polyvinylidene fluoride (Pvdf) electrospun fibers doped by carbon flakes. Polymers.

[41] Sobola D., Kaspar P., Částková K., Dallaev R., Papež N., Sedlák P., Trčka T., Orudzhev F., Kaštyl J., Weiser A. (2021). PVDF fibers modification by nitrate salts doping. Polymers.

[42] Sedlak P., Sobola D., Gajdos A., Dallaev R., Nebojsa A., Kubersky P. (2021). Surface analyses of PVDF/NMP/[EMIM][TFSI] solid polymer electrolyte. Polymers.

[43] Smejkalová T., Ţǎlu Ş., Dallaev R., Částková K., Sobola D., Nazarov A. (2021). SEM imaging and XPS characterization of doped PVDF fibers. E3S Web Conf..

[44] Kerbow D.L., Scheirs J. (1997). Modern Fluoropolymers.

[45] Cheng Y., Li D. (2020). Numerical analysis of piezoelectric signal of PVDF membrane flapping wing in flight. IOP Conf. Ser. Mater. Sci. Eng..

[46] Holmes-Siedle A.G., Wilson P.D., Verrall A.P. (1983). PVdF: An electronically-active polymer for industry. Mater. Des..

[47] Liu R., Yuan B., Zhong S., Liu J., Dong L., Ji Y., Dong Y., Yang C., He W. (2021). Poly(vinylidene fluoride) separators for next-generation lithium based batteries. Nano Select.

[48] Shabanov V.A., Konnov E.I. Sensing elements based on PVDF films for creating hydroacoustic transducers. Proceedings of the 2nd Youth Scientific Conference “Actual problems of piezoelectric instrument making”.

[49] Liu T., Zhou X., Sun Y., Bai R. (2021). Anticorrosion performance of pvdf membranes modified by blending ptfe nanoemulsion and prepared through usual non-solvent-induced phase inversion method. Membranes.

[50] Ghazali N., Basirun W.J., Nor A.M., Johan M.R. (2020). Super-amphiphobic coating system incorporating functionalized nano-Al_2_O_3_ in polyvinylidene fluoride (PVDF) with enhanced corrosion resistance. Coatings.

[51] Hussein A.A., Dawood N.M., Al-Kawaz A.E. (2021). Corrosion protection of 316L stainless steel by (PVDF/HA) composite coating using a spinning coating technique. Bull. Pol. Acad. Sci. Tech. Sci..

[52] Chakradhar R.P., Prasad G., Bera P., Anandan C. (2014). Stable superhydrophobic coatings using PVDF-MWCNT nanocomposite. Appl. Surf. Sci..

[53] Burkhart M., Wermelinger J., Setz W., Müller D. (1996). Suitability of polyvinylidene fluoride (PVDF) piping in pharmaceutical ultrapure water applications. PDA J. Pharm. Sci. Technol..

[54] Yessari M., Fangachi N., Rguiti M., Hajjaji A. (2022). Design and numerical simulation of a piezoelectric harvester using PVDF polymer for keyboard application. Mater. Today Proc..

[55] Klinge U., Klosterhalfen B., Birkenhauer V., Junge K., Conze J., Schumpelick V. (2002). Impact of polymer pore size on the interface scar formation in a rat model. J. Surg. Res..

[56] Klosterhalfen B., Klinge U., Schumpelick V. (1998). Functional and morphological evaluation of different polypropylene-mesh modifications for abdominal wall repair. Biomaterials.

[57] Klinge U., Klosterhalfen B., Müller M., Öttinger A.P., Schumpelick V. (1998). Shrinking of polypropylene mesh in vivo: An experimental study in dogs. Eur. J. Surg..

[58] Sukovatykh B.S., Netyaga A.A., Zhukovsky V.A., Valuyskaya N.M., Korovicheva S.Y. The up to date polymer materials in plastic surgery of postoperative and recurrent ventral hernias. Modern methods of surgical treatment of ventral abdominal hernias and eventrations. Kursk scientific and practical bulletin “Man and his health”, 2006, No.1. https://cyberleninka.ru/article/n/setchatye-implantaty-iz-polivinilidenftorida-v-lechenii-gryzh-bryushnoy-stenki/viewer.

[59] Sedov V.M., Tarbaev S.D., Rostovskoy A.A., Gorelov A.A. Surgical treatment of postoperative ventral hernias using polypropylene and PVDF mesh implants. Proceedings of the 5th International Conference Modern Approaches to the Development and Clinical Use of Effective Dressings, Suture Materials and Polymeric Implants.

[60] Jansen P.L., Klinge U., Anurov M., Titkova S., Mertens P.R., Jansen M. (2004). Surgical mesh as a scaffold for tissue regeneration in the esophagus. Eur. Surg. Res..

[61] Junge K., Rosch R., Klinge U., Krones C., Klosterhalfen B., Mertens P.R., Lynen P., Kunz D., Preiß A., Peltroche-Llacsahuanga H. (2005). Gentamicin supplementation of polyvinylidenfluoride mesh materials for infection prophylaxis. Biomaterials.

[62] Klinge U., Klosterhalfen B., Öttinger A.P., Junge K., Schumpelick V. (2002). PVDF as a new polymer for the construction of surgical meshes. Biomaterials.

[63] Lazarenko V.A. (2002). The choice of suture material for vascular plasty. Int. Congr. Surg. Petrozavodsk.

[64] Bezhin A.I., Dolzhikov A.A., Zhukovsky V.A., Netyaga A.A., Plotnikov R.V. (2007). Experimental substantiation of the use of new polyvinylidene fluoride endoprostheses with carbine coating for hernioplasty. Bull. New Med. Technol..

[65] Lee M., Catsouras I., Asadi K., Blom P.W.M., de Leeuw D.D. (2013). Low voltage extrinsic switching of ferroelectric δ-PVDF ultra-thin films. Phys. Lett..

[66] Ma H., Jen A.Y., Dalton L.R. (2002). Polymer-based optical waveguides: Materials, processing, and devices. Adv. Mater..

[67] Iwamoto N., Johnston R.W., Yokoi K., Nakano K., Fujita K., Misaki S., Sugimoto M., Johnston R.W., Kanazawa K., Misaki Y. Respiration and Heartbeat Signal Measurement with A Highly Sensitive PVDF Piezoelectric Film Sensor. Proceedings of the Second International Conference on Electronics and Software Science (ICESS2016).

[68] Hu X., You M., Yi N., Zhang X., Xiang Y. (2021). Enhanced Piezoelectric Coefficient of PVDF-TrFE Films via In Situ Polarization. Front. Energy Res..

[69] Kalimuldina G., Turdakyn N., Abay I., Medeubayev A., Nurpeissova A., Adair D., Bakenov Z. (2020). A Review of Piezoelectric PVDF Film by Electrospinning and Its Applications. Sensors.

[70] Chen W.W., An Z.L., He L.B., Deng Z. Piezoelectric coefficients measurement for PVDF films with pneumatic pressure rig in a sole cavity. Proceedings of the 2015 Symposium on Piezoelectricity, Acoustic Waves and Device Applications.

[71] Xu F., Chu F., Trolier-McKinstry S. (1999). Longitudinal piezoelectric coefficient measurement for bulk ceramics and thin films using pneumatic pressure rig. J. Appl. Phys..

[72] Kholkin A.L., Wütchrich C., Taylor D.V., Setter N. (1996). Interferometric measurements of electric field-induced displacements in piezoelectric thin films. Rev. Sci. Instrum..

[73] Lu K., Huang W., Guo J., Gong T., Wei X., Lu B.-W., Liu S.-Y., Yu B. (2018). Supersensitive strain gauge based on flexible poly(vinylidene fluoride) piezoelectric film. Nanoscale Resolut..

[74] Furlan A.D., Brosso L., Imamura M., Irvin E. (2002). Massage for low back pain: A systematic review within the Cochrane Collaboration Back Review Group. Spine.

[75] Shirafuji S., Hosoda K. Slip detection and prevention using sensors with different properties embedded in elastic artificial leather based on previous experience. Proceedings of the International Conference on Advanced Robotics.

[76] Liao H., Ava L., Nicola R. (2011). The Evidence for Shiatsu: A Systematic Review of Shiatsu and Acupressure. BMC Complementary Altern. Med..

[77] Dmitriev I.Y. (2007). Electroactive polymer systems based on porous films of polyvinylidene fluoride: Dissertation of a candidate of physical and mathematical sciences. Master’s Thesis.

[78] Korobova Y.G., Babaev V.G., Khvostov V.V., Guseva M.B. (2008). Emission characteristics of fibers based on linear chain carbon. Vestn. Mosc. Univ. Ser. 3 Phys. Astron..

[79] Mavrinskaya N.A., Pesin L.A., Baumgarten M., Mavrinskiy A.V., Baitinger E.M., Evsyukov S.E. (2008). ESR studies of chemically dehydrofluorinated poly(vinylidene fluoride). Magn. Reson. Solids. EJ.

[80] Evsyukov S.E., Kudryavtsev Y.P., Korshak Y.V. (1991). Chemical dehydrohalogenation of halogenated polymers. Russ. Chem. Rev..

[81] Kudryavtsev Y.P., Evsyukov S.E., Guseva M.B. (2010). Karbin—The Third Allotropic Form of Carbon. Nanotechnologies: Dev. Appl. XXI Century.

[82] Sencadas V., Moreira V.M., Lanceros-Mendéz S., Pouzada A.S., Gregório R. (2006). α- to -β Transformation On Pvdf Films Obtained By Uniaxial Stretch. Mater. Sci. Forum.

[83] Makarevich N.I., Sushko N.I. (1965). IR spectra and crystalline modifications of IR-polyvinylidene fluoride. Zh. Butt. Spectrosc..

[84] Semochkin P.S., Andreychuk V.P., Pesin L.A., Evsyukov S.E., Koryakova O.V., Belenkov E.A., Shakhova I.V. (2009). Effect of Uniaxial Tension on Phase Transformations of Polyvinylidene Fluoride Films. Bull. South Ural. State University. Ser. Mathematics. Mechanics. Phys..

[85] Vointseva I.I., Gil’man L.M., Kudryavtsev Y.P., Evsyukov S.E., Pesin L.A., Gribov I.V., Moskvina N.A., Khvostov V.V. (1996). Chemical Dehydrochlorination of Polytrichlorobutadienes. A New Route to Carbines. Europ. Polym. J..

[86] Kochervinsky V.V. (1996). Structure and properties of block polyvinylidene fluoride and systems based on it. Adv. Chem..

[87] Duca M.D., PLoSceanu C.L., Pop T. (1998). Effect of X-rays on Poly (Vinylidene Fluoride) in X-ray Photoelectron Spectroscopy. J. Appl. Polym. Sci..

[88] Pesin L.A., Gribov I.V., Kuznetsov V.L., Evsyukov S.E., Moskvina N.A., Margamov I.G. (2003). In Situ Observation of the Modification of Carbon Hybridization in Poly (Vinylidene Fluoride) during Xps/Xaes Measurements. Chem. Phys. Lett..

[89] Brzhezinskaya M.M., Morilova V.M., Baitinger E.M., Evsyukov S.E., Pesin L.A. (2014). Study of Poly (Vinylidene Fluoride) Radiative Modification Using Core Level Spectroscopy. Polym. Degrad. Stab..

[90] Sidelnikova A.L., Andreichuk V.P., Pesin L.A., Evsyukov S.E., Gribov I.V., Moskvina N.A., Kuznetsov V.L. (2014). Kinetics of Radiation-Induced Degradation of Cf2- And Cf-Groups in Poly (Vinylidene Fluoride): Model Refinement. Polym. Degrad. Stab..

[91] le Moël A., Duraud J.P., Balanzat E. (1986). Modifications of Polyvinylidene Fluoride (Pvdf) Under High Energy Heavy Ion, X-ray and Electron Irradiation Studied by X-ray Photoelectron Spectroscopy. Nucl. Instrum. Methods Phys. Res. B.

[92] Le Moël A., Duraud J.P., Lemaire I., Balanzat E. (1987). 1.; Ramillon, J.M.; Darnez, C. Electronic and Structural Modifications of Polyvinylidene Fluoride under High Energy Oxygen Ion Irradiation. Nucl. Instrum. Methods Phys. Res. B.

[93] le Moël A., Duraud J.P., Lecomte C., Valin M.T., Henriot M., le Gressus C., Darnez C., Balanzat E., Demanet C.M. (1988). Modifications Induced in Polyvinylidene Fluoride by Energetic Ions. Nucl. Instrum. Methods Phys. Res. B.

[94] Adem E.H., Bean S.J., Demanet C.M., le Moel A., Duraund J.P. (1988). Xps As A Tool For The Investigation of Polymers Irradiated By Energetic Ions. Nucl. Instrum. Methods Phys. Res. B.

[95] Pesin L.A., Morilova V.M., Zherebtsov D.A., Evsyukov S.E. (2013). Kinetics of Pvdf Film Degradation under Electron Bombardment. Polym. Degrad. Stab..

[96] Zhang S., Shen J., Qiu X., Wend D., Zhu W. (2006). ESR and Vibrational Spectroscopy Study on Poly (Vinylidene Fluoride) Membranes with Alkaline Treatment. J. Power Sources.

[97] Volegov A.A., Pesin L.A., Margamov I.G., Evsyukov S.E., Koryakova O.V., Kochedykov V.A. (2006). Evaluation of the depth and rate of penetration of a dehydrofluorinating mixture into polyvinylidene fluoride using IR spectroscopy. Proc. Chelyabinsk Sci. Cent..

[98] Ross G.J., Watts J.F., Hill M.P., Morrissey P. (2000). Surface Modification of Poly (Vinylidene Fluoride) by Alkaline Treatment 1. the Degradation Mechanism. Polymer.

[99] Ross G.J., Watts J.F., Hill M.P., Morrissey P. (2001). Surface Modification of Poly (Vinylidene Fluoride) by Alkaline Treatment. Part 2. Process Modification by the Use of Phase Transfer Catalysts. Polymer.

[100] Zhivulin V.E., Zherebtsov D.A., Pesin L.A. (2018). Molecular structure of chemically carbonized films of polyvinylidene fluoride (according to IR spectroscopy). Bull. Tomsk. Polytech. Univ. Eng. Georesources.

[101] Zhivulin V.E., Pesin L.A., Morilova V.M., Koryakova O.V. (2014). Influence of heat treatment on the magnetic activity of the products of chemical carbonization of polyvinylidene fluoride. Bull. Juurgu Ser. Math. Mech. Phys..

[102] Zhivulin V.E., Pesin L.A., Mezhenina O.A., Kovalev I.N., Zlobina N.A., Gavrilov M.A., Morilova V.M., Koryakova O.V. (2014). Influence of the duration of isothermal holding on the magnetic and structural properties of the products of chemical carbonization of polyvinylidene fluoride. Proc. Tomsk. Polytech. Univ. Math. Mech. Phys..

[103] Zhivulin V.E., Pesin L.A., Ivanov D.V. (2016). Peculiarities of temperature dependence of EPR absorption of chemically carbonized derivatives of polyvinylidene fluoride. Solid State Phys..

[104] Mavrinskaya N.A., Pesin L.A., Baumgarten M., Baitinger E.M., Mavrinsky A.V., Evsyukov S.E. (2008). Optical properties and EPR absorption of chemically dehydrofluorinated polyvinylidene fluoride. 123 Bull. Juurgu Ser. Math. Phys. Chem..

[105] Mavrinskaya N.A., Mavrinsky A.V., Baumgarten M., Baitinger E.M., Evsyukov S.E., Pesin L.A. (2008). Influence of conditions and duration of storage on the intensity of the EPR signal of chemically dehydrofluorinated derivatives of polyvinylidene fluoride. Bull. Juurgu Ser. Math. Phys. Chem..

[106] Kudryavtsev Y.P., Evsyukov S.E., Babaev V.G. (1992). Effective dehydrofluorinating system for polyvinylidene fluoride. Proc. Acad. Sci. Chem. Ser..

[107] Gordon A., Ford R. (1976). A Companion to Chemistry.

[108] Korshak V.V., Kudryavtsev Y.P., Korshak Y.V., Evsyukov S.E., Litovchenko G.D. (1987). Dehydrofluorination of polyvinylidene fluoride in the presence of tetrahydrofuran. Rep. Acad. Sci. USSR.

[109] Pesin L.A., Chebotarev S.S., Kuvshinov A.M., Bespal I.I., Gribov I.V., Moskvina N.A., Kuznetsov V.L., Evsyukov S.E., Vyazovtsev A.V., Kravets N.S. (2010). Peculiarities of electron emission spectra of products of radiation carbonization of polyvinylidene fluoride. Surf. X-Ray Synchrotron Neutron Res..

[110] Voinkova L.A., Pesin A.A., Volegov A.A., Evsyukov S. (2007). Depth distribution of fluorine concentration during radiation carbonization of PVDF. Surf. X-Ray Synchrotron Neutron Stud..

[111] Kochervinsky V.V. (2003). Structural aspects of piezoelectricity in crystallizing ferroelectric polymers on the example of homopolymer and copolymers of vinylidene fluoride. VMS Ser. B.

[112] Kochervinskii V.V. (1999). Ferroelectricity of polymers based on vinylidene fluoride. Russ. Chem. Rev..

[113] Kochervinskii V.V. (1994). The properties and applications of fluorine-containing polymer films with piezo- and pyro-activity. Russ. Chem. Rev..

[114] Wang T.T., Herbert J.M. (1988). The Application of Ferroelectric Polymers.

[115] Kochervinskii V.V. (1996). The structure and properties of block poly(vinylidene fluoride) and systems based on it. Russ. Chem. Rev..

[116] Kochervinsky V.V. (2003). Piezoelectricity in crystallizing ferroelectric polymers by the example of polyvinylidene fluoride and its copolymers. Crystallography.

[117] Ohigashi H. (1976). Electromechanical properties of polarized polyvinylidene fluoride films as studied by the piezoelectric resonance method. J. Appl. Phys..

[118] Sussner H. (1976). Physical interpretation of the anisotropy and temperature dependence of the piezoelectric constant of polyvinylidene fluoride. Phys. Lett. A.

[119] Furukawa T., Aiba J., Fukada E. (1979). Piezoelectric relaxation in polyvinylidene fluoride. J. Appl. Phys..

[120] Kochervinsky V.V., Sokolov V.G., Zubkov V.M. (1991). Influence of the molecular structure on the characteristics of the electrical hysteresis of polyvinylidene fluoride and its copolymers. High Mol. Weight. Compd. A.

[121] Scheinbeim J.J., Chung K.T., Rae C.D., Newman B.A. (1979). The dependence of the piezoelectric response of poly (vinylidene fluoride) on phase-I volume fraction. J. Appl. Phys..

[122] Nix E.L., Holt L., Mcgrath J.C., Ward I.M. (1981). Highly drawn poly (vinylidene fluoride) with enhanced mechanical and electrical properties. Ferroelectrics.

[123] Tasaka S., Niki J., Ojio T., Miyata S. (1984). Structure and Piezoelectricity of Poly (vinylidene fluoride) Films Obtained by Solid-State Extrusion. Polym. J..

[124] Wang T.T. (1979). Piezoelectricity in β-phase poly (vinylidene fluoride) having a “single-crystal” orientation. J. Appl. Phys..

[125] Nagai M., Uehara H., Kanamoto T. (1996). Drawing of poly (vinylidene fluoride): Effects of initial morphology and technique on the structure and properties of drawn products. Kobunshi Ronbunshu.

[126] Nagai M., Nakamura K., Uehara H., Kanamoto T., Takahashi Y., Furukawa T. (1999). Enhanced electrical properties of highly oriented poly (vinylidene fluoride) films prepared by solid-state coextrusion. J. Polym. Sci. Polym. Phys..

[127] Ibragimova A.I., Zhuravleva I.I., Kuznetsov S.I., Panin A.S., Tarasova E.Y. (2019). Structure and phase composition of polyvinylidene fluoride films obtained by laser synthesis. Bull. Lebedev Phys. Inst..

[128] Wachtler M., Wagner M.R., Schmied M., Winter M., Besenhard J.O. (2001). The Effect of the Binder Morphology on the Cycling Stability of LiAlloy Composite Electrodes. J. Electroanal. Chem..

[129] Yoo M., Frank C.W., Mori S., Yamaguchi S. (2004). Interaction of Poly(Vinylidene Fluoride) with Graphite Particles. 2. Effect of Solvent Evaporation Kinetics and Chemical Properties of Pvdf on the Surface Morphology of a Composite Film and Its Relation to Electrochemical Performance. Chem. Mater..

[130] Sedov V.M., Gostevskoy A.A., Tarbaev S.D., Gorelov A.S., Chulkhovin A.B., Nutfullina G.M., Zhukovsky V.A. (2008). Polyvinylidene fluoride mesh implants in the treatment of hernias abdominal wall. Surg. Her..

[131] Egiev V.N., Voskresensky P.K., Emelyanov S.I. (2002). Tension-Free Hernioplasty.

[132] Eremeev V.P., Rekhachev V.P., Kivermna Z.I. (1984). Treatment of postoperative ventral hernia. Surg. Her..

